# The Taurine-Slc6a6 Axis Promotes Breast Cancer Progression by Alleviating Oxidative Stress and Accelerating Cell Cycle Progression

**DOI:** 10.3390/cells15020207

**Published:** 2026-01-22

**Authors:** Pei Cai, Xiaoqin Liu, Meizhen Lin, Shaofang Xie, Lei Yuan, Zuobao Lin, Yue Zhang, Shang Cai

**Affiliations:** 1 Westlake Institute for Advanced Study, Fudan University, Shanghai 310018, China; caipei@westlake.edu.cn (P.C.);; 2 School of Life Sciences, Westlake University, Hangzhou 310024, China; 3 Westlake Laboratory of Life Sciences and Biomedicine, School of Life Sciences, Westlake University, Hangzhou 310024, China; 4 College of Life Sciences, Zhejiang University, Hangzhou 310024, China; 5 Westlake Disease Modeling Laboratory, Westlake Laboratory of Life Sciences and Biomedicine, Hangzhou 310024, China

**Keywords:** Taurine, Slc6a6, breast cancer

## Abstract

Taurine metabolism is emerging as an important player in cancer progression, yet its precise roles remain incompletely understood. Our study revealed that elevated serum Taurine levels and concomitant upregulation of its transporter, Slc6a6, are associated with enhanced tumor growth. Functionally, Slc6a6 overexpression drives tumor progression in vivo and accelerates cancer cell proliferation in vitro. Mechanistically, we identified a dual pro-oncogenic function for Slc6a6. First, Slc6a6 possesses intrinsic antioxidant regulatory capacity and further enhances cellular redox homeostasis by mediating the uptake of the antioxidant molecule Taurine. Second, beyond its metabolic role, Slc6a6 directly interacts with the cell cycle regulator Rprd1b to promote the G1/S phase transition, leading to uncontrolled proliferation. Clinically, bioinformatics analyses correlate high SLC6A6 expression with poor prognosis in breast cancer patients, underscoring its potential as a therapeutic target.

## 1. Introduction

Taurine is a sulfur-containing non-protein amino acid existing in almost all tissues of animals. Taurine exists in free form in vivo and does not participate in the biosynthesis of proteins [1,2,3,4]. This amino acid plays a role in various biological activities, including osmoregulation, membrane stabilization, antioxidation, bile salt formation, neurotransmission, and participation in cellular immunity [5,6,7,8]. Additionally, Taurine can be obtained from the diet and is taken up by cells through Taurine transporters [9,10]. Notably, Taurine deficiency is a driver of aging and affects healthy life span [9,10,11,12]. Moreover, Taurine has been implicated in tumor biology and has been found to have both pro-tumorigenic and anti-tumorigenic effects [13,14,15,16,17,18]. On the pro-tumor side, Taurine has been shown to promote tumor in certain types of cancers [19,20,21]. For example, it may act as an osmoregulator in tumor cells, helping them adapt to the hypertonic tumor microenvironment and survive under stress conditions [22,23]. Taurine and proline was found to be able to suppress Azgp1 expression and activate mTOR expression to promote lung cancer progression [24]. Conversely, Taurine has been declared to exhibit anti-tumorigenic effects as well [14,25,26,27]. It can induce apoptosis (programmed cell death) in cancer cells, inhibit tumor cell growth, and enhance the effectiveness of certain chemotherapeutic drugs [28,29].

The Taurine transporter TAUT, encoded by the *SLC6A6* gene, is responsible for the transport of Taurine, an amino acid, across cell membranes [30]. Building on the aforementioned links between Taurine and cancer, several studies have suggested that SLC6A6 plays an important role in tumorigenesis [31,32,33,34]. However, comprehensive analyses of SLC6A6 expression and its relationship with survival have not been performed in breast cancer. Regarding its function in tumors, the role of SLC6A6/TAUT is not well understood or extensively studied. Overall, the function of SLC6A6/TAUT in tumors is complex and context-dependent, and more research is needed to fully understand its role in tumor biology and its potential as a therapeutic target.

In this study, we identified the Taurine transporter Slc6a6 as a molecular determinant involved in breast cancer progression. First, we observed that tumor-bearing mice exhibited elevated serum Taurine levels alongside increased expression of the Taurine transporter Slc6a6. Genetic knockdown of Slc6a6 significantly suppressed cell proliferation, whereas its overexpression enhanced Taurine uptake, leading to decreased apoptosis and increased proliferation in cancer cells with enhancing the progression of the G1/S transition of cell cycle. Mechanistically, on the one hand, Slc6a6 acts as a central regulator of antioxidant defense via both intrinsic regulatory mechanisms and taurine-dependent pathways to reduce intracellular reactive oxygen species (ROS), thereby protecting cancer cells from oxidative damage; on the other hand, Slc6a6 directly interacts with the cell cycle regulator Rprd1b to accelerate the cell cycle. This dual mechanism positions Slc6a6 as a mediator of metabolic adaptation by coordinating Taurine uptake and cell cycle regulation. Furthermore, bioinformatics analysis of clinical datasets revealed that high SLC6A6 expression correlates with poor prognosis in certain subtype breast cancer patients. Our findings may provide novel targets and approaches for developing new therapies for breast cancer.

## 2. Materials and Methods

### 2.1. Non-Targeted Metabolomics

Blood samples were collected from wild-type (WT) and tumor-bearing mice via the orbital sinus/vein under anesthesia. The blood was transferred into lithium-heparin-coated tubes, gently inverted to mix, and then centrifuged at 3000 rpm for 10 min at room temperature. A 100 μL aliquot of the supernatant (serum) was carefully transferred into 1.5 mL Eppendorf tube, and then extracted with 80% methanol (pre-chilled to −80 °C). After being dried, they were stored at −80 °C for subsequent analysis. For mass spectrometry analysis, using 100 μL of an acetonitrile/water solution (1:1, *v*/*v*) to reconstitute the dried supernatant, vortexed, and centrifuged at 14,000× *g* for 15 min at 4 °C. The supernatant was then utilized for subsequent analysis. For quality control (QC), 10 μL of supernatant from each sample was pooled to prepare the QC samples. Metabolite analysis was conducted using an LC-MS system comprising an Agilent 1290 Infinity II UHPLC system tandem (Agilent, Santa Clara, CA, USA) with Bruker Tims TOF Pro2 (Bruker, Bremen, Germany). Chromatographic separation was achieved on an ACQUITY UPLC BEH Amide column (100 mm × 2.1 mm, 1.7 μm). The mobile phase consisted of 15 mM ammonium acetate, 0.3% NH_3_·H_2_O in water and 15 mM ammonium acetate, 0.3% NH_3_·H_2_O in 9:1 acetonitrile/water (B) at a flow rate of 0.3 mL/min. The column was eluted with 95% mobile phase B for 1 min, followed by a linear gradient to 50% mobile-phase B over 8 min, held at 50% for 3 min, a linear gradient to 95% mobile phase B over 0.5 min, then 1.5 min at 95% mobile-phase B. The sample volume injected was 2 μL. MS data were acquired using electrospray ionization in both positive and negative ion modes over 20–1300 *m*/*z*. The TIMS Setting include: 1/k0 from 0.1 V·S/cm^3^ to 1.5 V·S/cm^3^, Ramp Time 100 ms, Accu.Time 100 ms. Other MS settings include capillary 4500 V, Nebulizer 2 bar, Dry Gas 8 L/min, Dry Temp 230 °C, sheath gas temperature 400 °C, sheath gas flow 4 L/min. Metabolic differential analysis was carried out by MetaboAnalyst (https://www.metaboanalyst.ca/MetaboAnalyst/home.xhtml, accessed on 31 August 2025).

### 2.2. Targeted Metabolomics Analysis (Taurine Detection)

A sample of 100 μL of blood plasma was collected from each mouse and transferred into a 1.5-mL microcentrifuge tube. 10 μL of internal solution (Taurine standard (MCE, San Diego, CA, USA, HY-B0351)) was added to each sample followed by 400 μL methanol. Samples were incubated at −20 °C for 1 h, then centrifuged for 30 min at maximum speed at 4 °C. The supernatant was transferred to a new tube and dried under N_2_ flow, then resuspended in 50 μL methanol and transferred to micro-inserts. All samples were conducted using an Agilent 1290 Infinity II UHPLC system tandem with Agilent 6495 mass spectrometer. All data were collected and processed using the QQQ Quantitative Analysis Software(version B.09.00). Chromatographic separation was achieved on an ACQUITY UPLC BEH Amide column (100 mm × 2.1 mm, 1.7 μm) column. The mobile phase consisted of 10 mM ammonium acetate, 0.2%FA in water and acetonitrile (B) at a flow rate of 0.3 mL/min. The column was eluted with 90% mobile phase B for 1 min, followed by a linear gradient to 50% mobile-phase B over 4 min, held at 50% for 3 min, a linear gradient to 90% mobile phase B over 0.1 min, then 3.9 min at 90% mobile-phase B. The sample volume injected was 2 μL. Mass spectrometer operating in positive ion mode using the following settings: gas temperature 200 °C, gas flow 14 L/min, Nebulizer 20 psi, Sheath gas temperature 350 °C, Sheath gas flow 11 L/min, Capillary 3000 V, Nozzle Voltage 1500 V. The quantitative analysis of all compounds was measured by multiple reaction monitoring (MRM) mode. The MRM transitions used were 126/44, collision energy was 21 eV.

### 2.3. RNA Isolation and Real-Time qPCR

RNA isolation and real-time qPCR were conducted following previously established protocols [35]. Total RNA was extracted from the tumor cell population after isolating the target cells using FACS sorting. TRIzol Reagent (Invitrogen, Carlsbad, CA, USA, 15596018) was used for the RNA extraction process. The extracted RNA represented a total of 5000 cells and was subsequently reverse transcribed into cDNA using the HiScript^®^ II 1st Strand cDNA Synthesis Kit (+gDNA wiper) (Vazyme, Nanjing, China, R233). The resulting cDNA was quantified using real-time PCR in combination with a 2x SYBR Green qPCR Master Mix kit (Biomake, Houston, TX, USA, B21203) on an Analytik Jena AG detection system (Jena, jena Qtower384G, SIA-PCR006). To determine the relative mRNA levels, the ΔCt method was employed. The obtained values were expressed relative to Actin. The primer sequences used during the PCR were listed in the respective primer table.

### 2.4. Tumor Tissue Processing

Mouse breast tumors were dissected and processed according to a previously published protocol [36]. In brief, tumors were minced manually and mechanically into small fragments and digested with 0.5 mg/mL collagenase (Worthington, OH, USA, LS004182) and 50 U/mL hyaluronidase (Worthington, OH, USA, LS002592) for 2 h at 37 °C, with gentle pipetting every 60 min. The digested tissue was subjected to pulse centrifugation at 1500 rpm in DMEM-F12 medium. The resulting pellet was resuspended in 5 mL of ACK lysing buffer (Beyotime, Shanghai, China, C3702-120mL) and incubated on ice for 5 min to lyse erythrocytes. Tumor cells were then dissociated with 5 mL of 0.25% trypsin–ethylenediaminetetraacetic acid (Gibco) for 3–5 min, followed by brief digestion with 0.1 mg/mL DNase I (Worthington, OH, USA, LS002139). The cell suspension was filtered through a 40 μm strainer to obtain a single-cell suspension. Finally, cells were counted, resuspended in appropriate medium, and used for subsequent staining and FACS analysis or cryopreserved for future use.

### 2.5. Cell Lines

HEK293T cells (ATCC: CRL-3216) was obtained from the American Type Culture Collection (ATCC). HEK293T was cultured in DMEM media with 10% FBS,100 U/mL penicillin-streptomycin under 5% CO_2_ at 37 °C. PyMT cells were cultured in Advanced DMEM with 1% FBS, EGF, R-spondin1, 100 U penicillin and Y-27632. All cells were regularly screened to be mycoplasma-free.

### 2.6. Western Blot Assays

Western blot assays were performed according to a previously described protocol [37,38]. Total protein was extracted from target cell lines using RIPA or NP-40 lysis buffer supplemented with protease and phosphatase inhibitors. A total of 30 μg of protein was loaded onto 8–12% SDS-PAGE gels for electrophoretic separation. Proteins were then transferred onto polyvinylidene difluoride (PVDF) membranes via electroblotting. Membranes were blocked with 5% skim milk in Tris-buffered saline containing 0.1% Tween-20 (TBST) and incubated overnight at 4 °C with the following primary antibodies: Slc6a6 (HUABIO, Hangzhou, China, #ER651000, 1:500), Slc6a6 (Abclonal, Wuhan, A14783,1:500), Actin (Proteintech, Wuhan, China, #66009-1-Ig, 1:2000), Lamin B1 (Proteintech, Wuhan, China, #12987-1-AP, 1:500) and Myc-tag (HUABIO, #R1208-1, 1:500). DYKDDDDK Tag (FLAG) Mouse Monoclonal Antibody (HUABIO, #M1403-2, 1:1000). Following three washes with TBST, membranes were incubated with horseradish peroxidase-conjugated secondary antibodies for 1 h at room temperature. Protein bands were detected using SuperSignal Femto Western Blot Substrate (Thermo Fisher Scientific, Waltham, MA, USA) and visualized using a GE AI680RGB gel imaging system.

### 2.7. Mice

Female MMTV-PyMT transgenic mice (FVB/N-Tg(MMTV-PyVT)634Mul/J, 002374), which spontaneously develop breast tumors, were purchased from The Jackson Laboratory (Bar Harbor, ME, USA). They were bred at the laboratory animal resources center of Westlake University. Female FVB mice were purchased from Shanghai SLAC Laboratory Animal Company and acclimated to the animal facility for one week prior to initiating experiments.

Approval for all mouse experiments was granted by the Westlake University Animal Ethics Committee, and all mice were bred and housed according to institutional guidelines. All animal experiments were conducted in accordance with the laws and regulations of China. The local institutional animal ethics board approved all mouse experiments (permission numbers: 19-001-CS).

### 2.8. Tumor Transplantation Assays

Tumor cells were suspended in a 1:1 mixture of growth factor-reduced Matrigel and PBS for subcutaneous transplantation into FVB mice. A total of 0.5–5 × 10^6^ cells were injected per site. The same cell number was used within each experiment, although different experiments may have used different cell types or quantities. Cells were injected subcutaneously into the flank region of anesthetized mice. Tumor growth was measured using caliper until tumors reached about 2 cm in diameter or until mice showed signs of distress. Tumor volume was calculated using the formula: tumor volume (mm^3^) = (D × d^2^)/2, where D is the longest diameter and d is the shortest perpendicular diameter.

### 2.9. Taurine Administration

Mice were administered Taurine orally through drinking water at a dose of 100 mg/kg body weight daily, starting after tumor inoculation and continuing on a daily basis thereafter. Control mice received plain drinking water without Taurine supplementation.

### 2.10. Immunofluorescence

For immunofluorescence assays, cells were cultured on glass-bottom cell culture dishes (NEST, Palo Alto, CA, USA, 801001) and fixed with 4% paraformaldehyde at 25 °C for 15 min. Cells were then permeabilized with 0.1% Triton X-100 in Tris-buffered saline (TBS) for 5 min and blocked with 5% donkey serum for 1 h at room temperature. Following blocking, cells were incubated with the indicated primary antibodies overnight at 4 °C. After two washes with TBS, cells were incubated with fluorescently labeled secondary antibodies (IgG) for 1 h at room temperature. Nuclei were counterstained with 100 ng/mL DAPI staining solution (Sigma-Aldrich, St. Louis, MO, USA, D9542) for 5 min. Fluorescence images were acquired using an Olympus IX73 microscope imaging system (Olympus, Tokyo, Japan) and processed further using Nikon NIS-Elements AR analysis software (version 5.01.00, 64-bit).

### 2.11. CCK8 Assay

A CCK-8 kit (Yeasen, Shanghai, China, 40203ES80) was used to evaluate cell proliferation. Briefly, 3000 cells were seeded into each well of a 96-well plate and cultured in 180 μL of medium. Cell proliferation was assessed at 0, 24 h, 48 h, and 72 h post-seeding according to the manufacturer’s protocol. At each time point, 20 μL of CCK-8 reagent was added to 180 μL of culture medium to yield a 200 μL reaction mixture. The cells were then incubated for 2 h, after which the absorbance at 450 nm was measured.

### 2.12. Cell Cycle

Cell cycle assay was determined by Cell Cycle Analysis Kit (Beyotime, C1052). The experimental procedure was carried out according to the manufacturer’s instructions. Briefly, the cells were harvested and fixed in pre-cooled 70% ethanol for overnight at 4 °C and then stained with a solution containing propidium iodide (0.05 mg/mL), RNase A (1 mg/mL), and 0.3% Triton X-100 in the dark for 30 min. The percentage of cells in different phases of the cell cycle was examined by measuring the DNA content (propidium iodide intensity) with a flow cytometer (Beckman, CytoFLEX, Duarte, CA, USA). Each experiment was independently repeated three independent times.

### 2.13. RNA Interference

Cells were transfected with the Rprd1b siRNAs or control siRNAs by Hieff Trans^®^ in vitro siRNA/miRNA Transfection Reagent (Yeasen, 40806ES0). The transfection was performed according to the manufacturer’s instructions. siRNA sequences are listed in primer table of Supplementary Materials.

### 2.14. Generation of Cell Lines Overexpression or shRNA of Target Genes

(1) To achieve overexpression of Slc6a6, it was cloned into a pCDH-CMV-MCS-EF1 vector by performing PCR on tumor cell cDNA by using 2X MultiF Seamless Assembly Mix (ABclonal, Wuhan, China, RK21020).

(2) As for Knockdown of Slc6a6, it was cloned into a pSIH-H1 vector by performing according to the manufacturer’s instructions (System Biosciences, Palo Alto, CA, USA, Cat. #s SI500A-1). The predesigned target sequences for Slc6a6 are listed in primer table. The scramble shRNA obtained from Addgene (Cambridge, MA, USA) was used as control. 48 h after viral infection, Slc6a6 knockdown was confirmed by qPCR analysis.

Lentivirus production involved transfecting the transfer plasmids, containing the sgRNA or ORF sequences, along with packaging plasmids (Addgene, Watertown, MA, USA, plasmid #12260 psPAX2, #12259 pMG2.G) into HEK293T cells using polyethylenimine (PEI) (Sigma-Aldrich, St. Louis, MO, USA, 408727) as the transfection reagent. After 24 h, the medium was refreshed. After 48 h and 72 h, the lentivirus-containing supernatant was collected and concentrated for transduction.

### 2.15. Flow Cytometry

For Cd200 single cell sorting for qPCR, the PyMT tumor single cells were stained with CD45 (Biolegend, San Diego, CA, USA, 1:200), CD31 (Biolegend, 1:200), Ter119 (Biolegend, 1:200), CD49f (Biolegend, 1:200), EpCAM (Biolegend, 1:200), Cd200 (Biolegend, 1:200) with appropriate conjugated fluorophores for 30 min on ice. Then cells were washed and resuspended in HBSS + 2%FBS + 1%P/S + DAPI (1 μg/mL) at a density of 10 million/mL. Then stained samples were analyzed and sorted on BD FACSAriaTM Fusion (BD Bioscience) with a 100-μm nozzle. and the analysis was conducted using FlowJo software V10.8.1.

### 2.16. Annexin V Test

Primary tumor cells were stained with APC-annexin V (BioLegend, 640920, 1:200) according to the manufacturer’s instructions. Approximately 5 × 10^5^ cells were seeded in 6-well plates and cultured in DMEM supplemented with or without 10 μM Taurine for 2 h, followed by treatment with 1 mM H_2_O_2_. After 24 h, cells were harvested and washed twice with cold PBS. Single-cell suspensions were washed with Binding Buffer (BB) and incubated with APC-annexin V in BB for 20 min at 37 °C. Following staining, a fivefold volume of BB containing DAPI was added. Samples were analyzed using a CytoFLEX flow cytometer, and data were processed with FlowJo software V10.8.1. Each experiment was performed in triplicate.

### 2.17. ROS Test

The MitoSOX Red dye (Thermo, M36008) was employed to assess mitochondrial reactive oxygen species (mtROS) levels, following the manufacturer’s instructions. After treatment and wash with PBS, the cells were stained with 5 µM Mitosox-Red in pre-warmed serum-free DMEM, shielded from light for 30 min at 37 °C and 5% CO_2_. Fluorescence was measured using an excitation/emission wavelength of 396 nm/610 nm by CytoFlow Cytometer, and the data was analyzed using Flowjo analysis. Each assay was repeated at least three times.

### 2.18. GSH Determination

Glutathione (GSH) levels were determined using the GSH/GSSG Assay Kit (Beyotime, S0053) according to the manufacturer’s instructions. Cells were pretreated with or without Taurine for 2 h, followed by exposure to H_2_O_2_ (1 mM) for an additional 2 h in 6-well plates at equal cell densities. GSH levels were then measured following the kit protocol.

### 2.19. Co-Immunoprecipitation Coupled with Mass Spectrometry (Co-IP/MS) Analyses

Co-immunoprecipitation coupled with mass spectrometry (Co-IP/MS) analyses were performed by a professional team, and the detailed methodology is described in the referenced study [39]. Briefly, a modified tandem affinity purification protocol combined with mass spectrometry (TAP/MS) was employed, involving a two-step purification process. This approach utilizes the S-, 2 × FLAG-, and Streptavidin-Binding Peptide (SBP) tandem tag system (SFB-tag) for efficient protein purification. The protocol enables the identification of protein interactors and facilitates the construction of a high-confidence protein–protein interaction network through computational modeling. It is particularly valuable for identifying bona fide interacting proteins for downstream functional characterization.

### 2.20. Co-Immunoprecipitation Validation

Plasmids encoding the indicated genes were amplified from PyMT cell cDNA via reverse transcription polymerase chain reaction (RT–PCR). All constructs were subcloned into vectors containing the specified SFB or Myc tags to enable expression of the corresponding fusion proteins. Primer sequences are provided in the primer table. For immunoprecipitation assays, 1 × 107 cells were lysed with NETN buffer on ice for 30 min. The lysates were then incubated with 20 μL of conjugated S-beads (for SFB-tagged pull-down) for 2 h at 4 °C. The beads were washed three times with NETN buffer and boiled in 2× loading buffer. The lysates were subjected to SDS–PAGE followed by WB. The following primary antibodies were used: Mouse Monoclonal Antibody (HUABIO, EM31105, 1:1000), ANTI-FLAG M2 antibody (DYKDDDDK Tag Antibody) (CST, 2368 S, 1:1000),The following secondary antibodies were used: HRP-Donkey anti mouse (CST, 7076 S, 1:10000), HRP-Donkey anti rabbit (CST, 7074 S, 1:1000) at RT for 1 hr. Membrane was subsequently washed 3 × 10 min by PBST and developed using SuperSignal^®^ West Dura Extended Duration Substrate (Thermo Scientific, Waltham, MA, USA, 34094) and imaged by Gel imaging system (GE, AI680RGB).

### 2.21. Molecular Docking Analysis

Since the X-ray crystal structure of mouse Rprd1b is incomplete and only exhibits the C-terminal structure, we retrieved the three-dimensional structures of Slc6a6 and Rprd1b from the UniProt database (proteins O35316_Slc6a6_MOUSE and Q9CSU0_Rprd1b_MOUSE) for molecular docking analysis. Docking was performed using the HDOCK program [40], which generated 100 candidate binding conformations ranked by a comprehensive scoring function. The conformation with the lowest docking score was selected as the final binding model. The resulting three-dimensional complex structure was visualized and rendered using PyMOL (Version 3.0.3) [41]. To analyze intermolecular interactions, LigPlot+ was used to generate two-dimensional interaction diagrams [42]. PyMOL employs geometric algorithms to detect and display hydrogen bonds and spatial conformations in three dimensions, while LigPlot+ produces two-dimensional representations based on predefined geometric and statistical criteria. Together, these tools provide complementary insights into the molecular interactions within the complex.

### 2.22. Statistics

The animal cohorts for the experiments consisted of the same strain, sex, and size, and were randomly assigned to different experimental groups. The statistical parameters can be found in the Figures. GraphPad Prism 9 software was used for the statistical analysis. In brief, a Student’s *t*-test was employed to compare distribution differences between two groups, while a one-way analysis of variance (ANOVA) was performed for comparisons involving more than two groups.

## 3. Results

### 3.1. Taurine Levels Are Elevated in Tumor-Bearing Mice, and Its Transporter, Slc6a6, Is Highly Expressed in the Tumor

**Figure 1:**
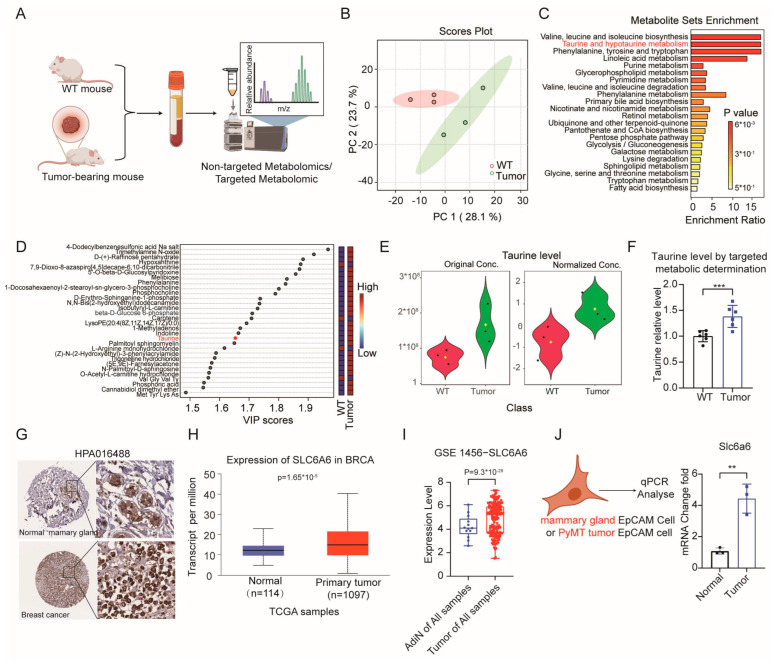
Taurine levels are elevated in tumor-bearing mice, and its transporter, Slc6a6, is highly expressed in the tumor. (**A**) Schematic representation of the workflow for serum metabolomics in wild-type (WT) or tumor-bearing mice. (**B**) Principal component analysis (PCA) of the expressed metabolites data, where each dot represents a unique sample. The PCA plot illustrates the separation between the wild-type and tumor-bearing groups. (**C**) Overview of KEGG pathway enrichment analysis based on metabolomic profiles of 33 blood biomarkers in wild-type mice (*n* = 3) and tumor-bearing mice (*n* = 3). (**D**) VIP score plot for identifying significant constituents (VIP score > 1) based on PLS-DA analysis. (**E**) Relative Taurine concentration in wild-type and tumor-bearing groups as determined by non-targeted metabolomics. (**F**) The concentration of Taurine in the blood of tumor-bearing mice was determined using the internal standard method (with Taurine reference substance). (*n* = 6 for each group). (**G**) Representative images of immunohistological staining for SLC6A6 expression in human normal mammary gland or breast cancer tissue from Human Protein Atlas (HPA) database with HPA016488 antibody, accessed on 11 August 2024. (**H**) Analysis of *SLC6A6* mRNA expression in tumor and adjacent non-tumor tissues using the TCGA breast cancer dataset from UALCAN database, accessed on 11 August 2024. (**I**) Analysis of SLC6A6 mRNA expression in tumor and adjacent non-tumor tissues using the GEO dataset GSE1456, accessed on 11 August 2024. (**J**) qPCR was used to analyze Slc6a6 expression in EpCAM-sorted mammary gland cells from normal and tumor-bearing mice. Data are presented as mean ± SD, ** *p* < 0.01, *** *p* < 0.001.

It is known that tumors undergo metabolic reprogramming [43,44,45], to investigate the differences in metabolites in the blood between cancer patients and healthy individuals, we conducted small molecule profiling in blood samples from both tumor-bearing and non-tumor-bearing mice on MMTV-PyMT breast cancer model, revealing significant alterations in various metabolites. The principal component analysis (PCA) of serum metabolite profiles demonstrated a significant difference between the WT mice and the tumor-bearing mice (Figure 1A,B). Notably, Taurine levels were significantly elevated in tumor-bearing mice (Figure 1C–E). This finding was confirmed by targeted Taurine measurements using Taurine standards, which confirmed higher Taurine concentrations in the tumor group compared to the WT group (Figure 1F and Supporting Figure S1).

Given that Taurine is transported by Slc6a6 in mice, we investigated whether Slc6a6 expression is upregulated in tumor-bearing mice. From the Human Protein Alts (HPA) database [46], we observed higher SLC6A6 protein levels in tumor tissues compared to normal mammary gland tissues (Figure 1G), suggesting that SLC6A6 may be upregulated in tumors. Consistently, bioinformatics analysis based on the TCGA database and GEO datasets GSE1456 from UALCAN database [47,48] revealed a significant upregulation of SLC6A6 in breast cancer tissues as compared to that in the matched normal tissues (Figure 1H,I). Quantitative PCR analysis of mammary tissues from normal mice and tumor tissues from tumor-bearing mice confirmed higher Slc6a6 expression in the latter group (Figure 1J).

Furthermore, our preliminary laboratory findings that the mammary aging process is asynchronous and progressive, initiated by an early senescence program, succeeded by an entropic late senescence program with elevated cancer associated pathways, vulnerable to cancer predisposition [37]. The transition towards senescence program is governed by a stem cell factor Bcl11b, loss of which accelerates mammary aging with enhanced DMBA-induced tumor formation. The paper revealed transcriptomic changes in both aged and young mice [37], with a notable increase in Slc6a6 protein expression observed in the mammary glands of mutant (Bcl11b loss) mice which displayed a mammary ageing phenotype (Supporting Figure S2A). Aging increases the incidence of tumors, and we observed high expression of Slc6a6 in the aging group; thus, this result provides additional support that Slc6a6 may be involved in tumorigenesis. Together, these findings suggested a potential link between elevated Slc6a6 and breast cancer.

### 3.2. Overexpression of SLC6A6 Reduces Apoptosis and Promotes Proliferation, Thereby Facilitating Tumor Growth

**Figure 2:**
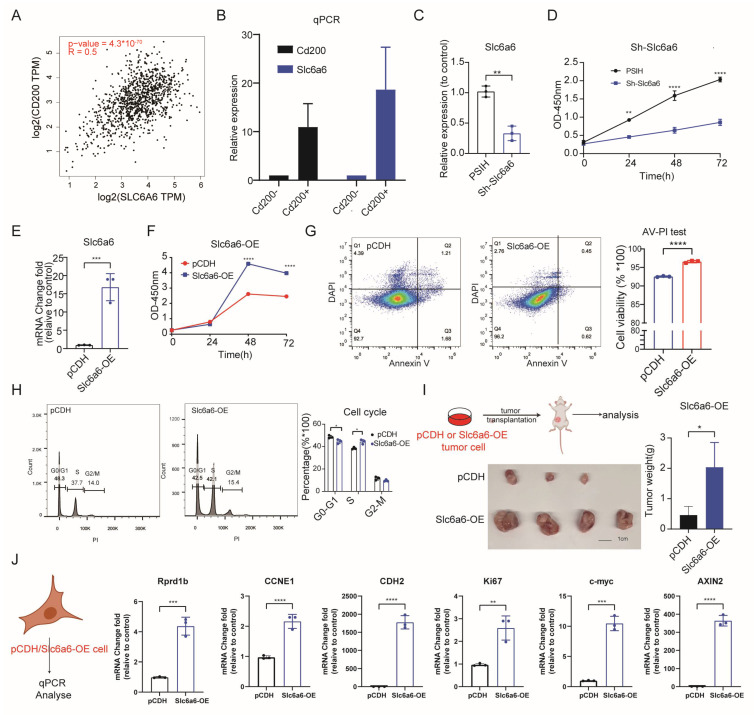
Overexpression of SLC6A6 reduces apoptosis and promotes proliferation, thereby facilitating tumor growth. (**A**) Pearson’s correlation found a positive correlation between CD200 expression and SLC6A6 by GEPIA (Gene Expression Profiling Interactive Analysis (http://gepia.cancer-pku.cn), accessed on 11 August 2024 (**B**) qPCR validated that Cd200+ cells represent cells with high expression of Slc6a6. (**C**) qPCR confirmed that knockdown of Slc6a6 protein effectively reduced the expression of Slc6a6 on Cd200+ tumor cell. (**D**) CCK-8 assay demonstrated that knockdown of Slc6a6 reduces cell viability. (**E**) qPCR confirmed that overexpression of Slc6a6 significantly increased the expression of Slc6a6 (**F**) The CCK-8 assay demonstrated that Slc6a6 overexpression (Slc6a6-OE) cells promoted cell proliferation. (**G**) Annexin V assay demonstrated that Slc6a6-OE cells exhibit reduced apoptosis and enhanced cell survival. (**H**) Pyridine iodide staining demonstrated that Slc6a6-OE cells promoted cell cycle. (**I**) Tumor growth in mice transplanted with Slc6a6-OE cells was significantly enhanced compared to that in mice transplanted with pCDH control cells (*n* = 3 for control group, *n* = 4 for Slc6a6-OE group). (**J**) qPCR analysis was performed to detect the effects of Slc6a6 overexpression on cell proliferation or cell growth related genes. Data are presented as mean ± SD, * *p* < 0.05, ** *p* < 0.01, *** *p* < 0.001, **** *p* < 0.0001.

Due to tumor heterogeneity, we adopted the PyMT tumor model, which better recapitulates tumor initiation and progression, to investigate the function of Slc6a6. Due to the lack of suitable flow cytometry antibodies for enriching and characterizing the Slc6a6 cell population, we attempted to identify alternative surface markers for enriching these cells. Our laboratory previously used Cd200 to label basal cells in the normal mammary gland, enabling the sub-fractionation of mammary basal cells based on Cd34 expression, with Cd200+ cells demonstrating enhanced clonogenic potential [49]. Differential Cd200 expression was observed across distinct structures within the mammary gland tissue, Many literature have identified Cd200, an immunosuppressive molecule [50,51,52], as a surface marker of tumor stem cells in various cancer types, including basal cell carcinoma, leukemia, and head and neck squamous cell carcinoma [53,54,55]. First, we evaluated the correlation between SLC6A6 and CD200 expression using data from The Cancer Genome Atlas (TCGA) database and got a positive correlation between SLC6A6 and CD200 expression (Figure 2A). Then, we propose whether Cd200+ cell can be utilized to enrich the Slc6a6+ cell population for further investigation. We first isolated Cd200+ cells via fluorescence-activated cell sorting (FACS) to enrich Slc6a6-expressing populations, and then confirmed through qPCR that cells with high Cd200 expression also exhibited higher Slc6a6 expression (Figure 2B). Subsequently, we expanded the Cd200+ cells in vitro and applied shRNA-mediated knockdown to examine the functional role of Slc6a6. We tested three shRNAs and confirming their efficiency, retained the most effective one line to expand the cell for subsequent experiments. qRT-PCR analysis confirmed that Slc6a6 was efficiently silenced in Cd200+ high expression cells (Figure 2C). CCK8 test showed that Slc6a6 knockdown markedly suppressed cell proliferation (Figure 2D). Next, we overexpressed Slc6a6 (Slc6a6-OE) in Cd200-PyMT cells by transfection with the constructed Slc6a6 overexpression plasmid. Slc6a6-OE cells exhibited a significant increase in cell proliferation compared to the control cells (Figure 2E,F). To further investigate Slc6a6 role in cell proliferation, we examined the effects of Slc6a6 on apoptosis and the cell cycle. Slc6a6-OE cells significantly reduced apoptosis and increase the cell viability by Annexin V staining (Figure 2G). Additionally, the fluorescent dye PI could enter cells having compromised membranes, and bind to DNA stoichiometrically, allowing evaluation of cell proportions in each phase based on their DNA levels. We could observe that Slc6a6-OE cells promoted the transition of cells from the G0/G1 phase to the S phase of the cell cycle, as evidenced by a significant decrease in the G0/G1 population and a concomitant increase in the S phase fraction gradually increased from 37% to 42% (Figure 2H). Similarly, we found higher cell cycle activity in the mammary glands of mutant (Bcl11b loss) mice which exhibited a mammary aging phenotype (Supporting Figure S2B). Moreover, we further evaluated the role of Slc6a6 in promoting breast cancer progression in vivo by injecting Slc6a6-OE cells into FVB mice. To minimize additional stress on mice during tumor size assessments, tumor growth curves were not monitored. Tumor weight was analyzed at the experimental endpoint. The results demonstrated that Slc6a6-OE significantly increased tumor weight compared to the control group (Figure 2I). Furthermore, we found that Slc6a6-OE significantly upregulates the expression of these related genes associated with cell proliferation and growth by qPCR (Figure 2J). Together, these data suggest Slc6a6 maybe serve as an oncogene in breast cancer progression.

### 3.3. Slc6a6 Promotes Tumor Growth by Acting as a Central Regulator of Antioxidant Defense via Both Intrinsic Regulatory Mechanisms and Taurine-Dependent Pathways

**Figure 3:**
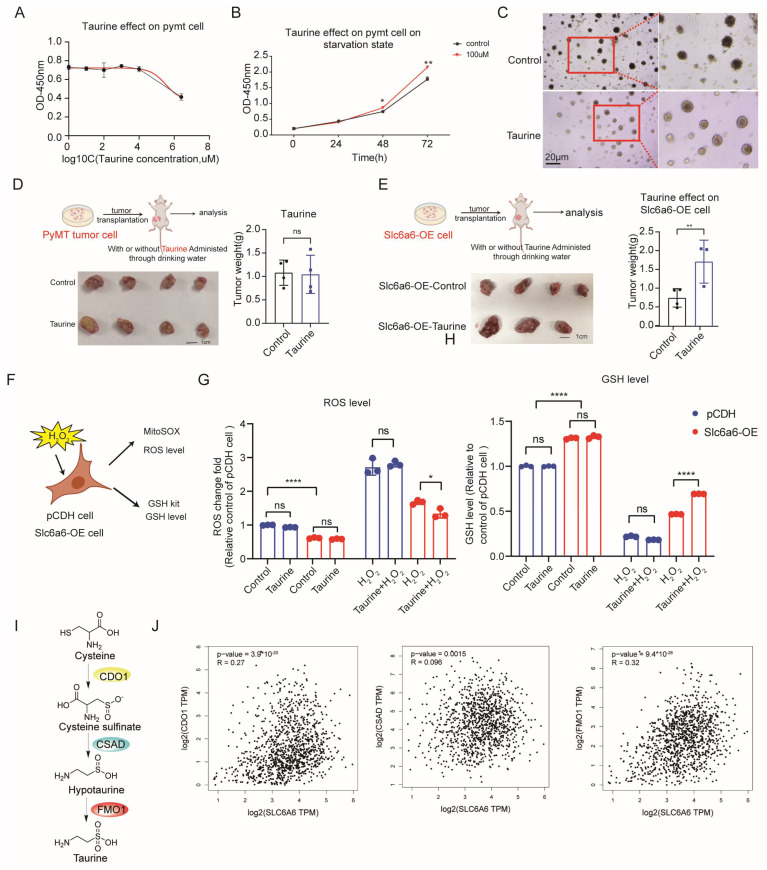
SLC6A6 promotes tumor growth by acting as a central regulator of antioxidant defense via both intrinsic regulatory mechanisms and taurine-dependent pathways. (**A**) The PyMT tumor cells can tolerate a wide range of high Taurine concentrations (0.001–10 mM) with relatively low toxicity for 24 h treatment. (**B**) Taurine promotes PyMT tumor cell proliferation under 100 μM for 72 h treatment. (**C**) Colony formation differed between conditions with and without Taurine treatment (100 μM). Sorted Cd200+ primary tumor cells (3000 per well) were seeded into 3D 96-well plates and cultured in Matrigel. After three days of colony growth, the medium was replaced with serum-free medium supplemented with Taurine (100 μM), and colony formation was monitored for eight days. The image shows the colonies on the final day of observation. (**D**) After implanting PyMT tumor cells into FVB mice, the animals were treated with or without Taurine (100 mg/kg) administered via drinking water; no difference in tumor growth was observed (*n* = 4 for each group). (**E**) Taurine promoted tumor growth with implanting Slc6a6-OE tumor cells into FVB mice. The animals were treated with or without Taurine (100 mg/kg) administered via drinking water (*n* = 4 for the control group and *n* = 3 for the Taurine group). (**F**) Schematic illustration of cellular ROS and GSH detection. (**G**) Taurine treatment enables Slc6a6-OE cells to reduce H_2_O_2_-induced ROS levels after 12 h post-exposure. The data was normalized with the basal condition control of pCDH group (*n* = 3 for each group). (**H**) Taurine treatment enables Slc6a6-OE cells to restore glutathione levels reduced by H_2_O_2_ exposure 2 h. The data was normalized with the basal condition control of pCDH group (*n* = 3 for each group). (**I**) Schematic illustration of the in vivo Taurine biosynthesis pathway, including three key enzymes CDO1, CSAD and FMO1 (CDO1 represents cysteine dioxygenase type 1, CSAD denotes cysteine sulfinic acid decarboxylase, FMO1 refers flavin containing dimethylaniline monooxygenase 1). (**J**) Correlation analysis between SLC6A6 expression and key enzymes involved in Taurine synthesis on breast cancer patient’s sample by GEPAI database with Spearman analysis, accessed on 9 October 2025. Data (G, H) are presented as mean ± SD, * *p* < 0.05, ** *p* < 0.01, **** *p* < 0.0001, ns, not significant.

To determine whether the impact of Slc6a6 on tumors is dependent on Taurine, given that current evidence indicates its primary function is Taurine transport. First, we stimulated cultured cells with Taurine and found that the cells tolerate Taurine over a broad concentration range, with apoptosis occurring only at concentrations as high as 20 mM (Figure 3A). However, reported Taurine levels in the human body are approximately 100 μM [9,31]. Previous studies have found that Taurine has a pro-apoptotic effect, which may be attributable to the use of concentrations inconsistent with physiological levels, thus representing an artifact [56,57]. Moreover, by using Taurine as a reference standard to measure the Taurine concentration in mouse blood, we found that its level was also approximately 100 μM (Supporting Figure S1). We initially treated tumor cells with Taurine for a long time and observed that Taurine enhanced cell proliferation, with a significant effect evident at 72 h, a milder effect at 48 h in a normal culture media condition (Figure 3B). To more accurately reflect the impact of Taurine on tumors, we performed a colony formation assay. Equal numbers of Cd200+ primary tumor cells were seeded into 3D 96-well plates and cultured on Matrigel. After colonies had grown for three days, the medium was replaced with serum-free medium supplemented with Taurine (100 μM), and colony formation was monitored. The results showed that Taurine significantly alleviated the reduction in colony size caused by nutrient deprivation. Specifically, colonies treated with Taurine exhibited larger sizes and better growth state, whereas control colonies showed signs of cell death and dispersion due to insufficient nutrients (Figure 3C), which is consistent with the CCK-8 assay results for Taurine promote cell proliferation (Figure 3B). Therefore, we administered Taurine via drinking water to mice to evaluate its effect on tumor growth following primary PyMT tumor implantation. Unfortunately, no differences in tumor growth were observed (Figure 3D). We hypothesize that the relatively low abundance of Slc6a6-positive cells in primary PyMT cells may limit the detectability of Taurine’s effects. To address this, we administered Taurine to xenografted mice bearing Slc6a6-OE tumor and found that Taurine promotes tumor growth (Figure 3E).

Next, we try to investigate whether this tumor-promoting effect is dependent on Taurine. Based on existing literature and the chemical structure of Taurine, we hypothesized that Taurine may reduce intracellular ROS levels [58,59,60]. To this end, we measured intracellular ROS and glutathione (GSH) levels. Our results showed that in cells with high Slc6a6 expression, Taurine significantly attenuated H_2_O_2_-induced ROS production and increased GSH levels. In contrast, control pCDH cells did not exhibit this protective effect (Figure 3F–H). Notably, under basal conditions without H_2_O_2_ challenge, Slc6a6-overexpressing cells exhibited significantly lower basal ROS levels and higher GSH levels compared to pCDH controls, indicating a constitutive antioxidant effect. This likely contributes to the observed basal viability advantage (Figure 2F). Collectively, Slc6a6 exerts a dual antioxidant role: it enhances the endogenous antioxidant program while simultaneously supplying exogenous Taurine to directly scavenge ROS. The above results suggest that Slc6a6 may enhance tumor cell survival and protect against ROS. Moreover, based on the TCGA database from the GEPAI database [61], we performed a correlation analysis between the expression of key enzymes involved in Taurine synthesis and SLC6A6. The results revealed a highly significant association between SLC6A6 and FMO1, the final rate-limiting enzyme in Taurine synthesis (Figure 3I,J), indicating that the impact of SLC6A6 on tumors is largely dependent on Taurine.

### 3.4. Slc6a6 May Promotes Tumor Cell Proliferation by Binding to Rprd1b

**Figure 4:**
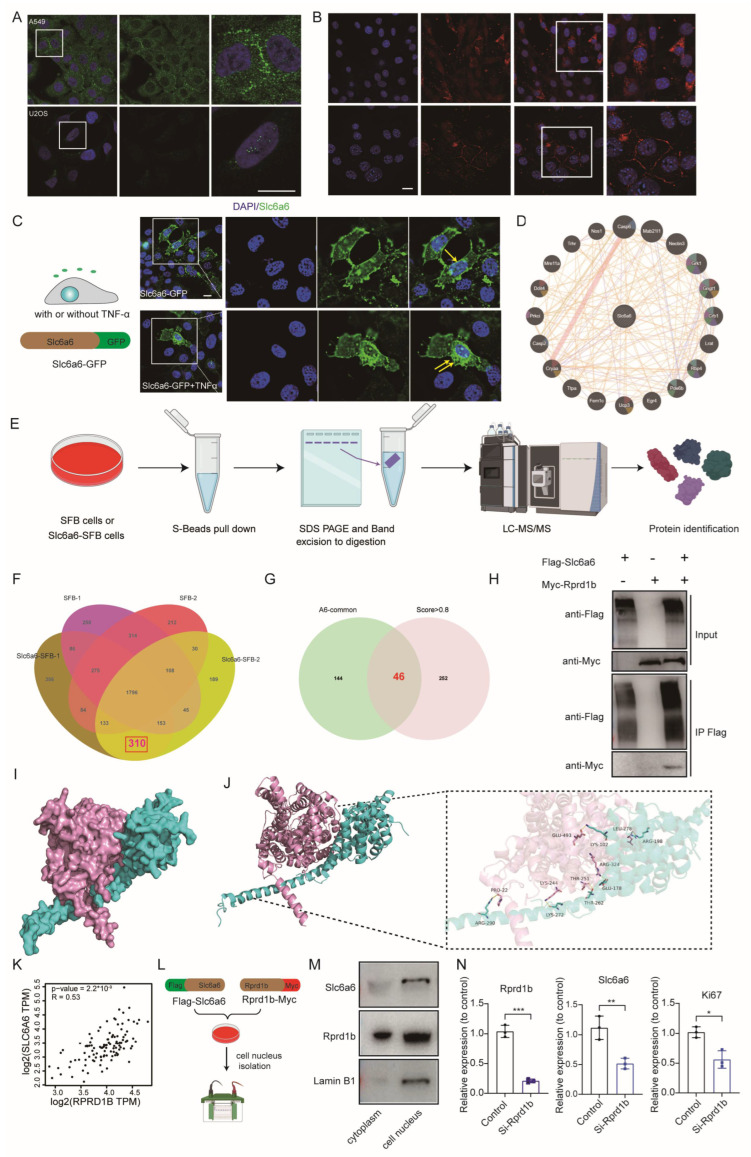
Slc6a6 may promotes tumor cell proliferation by binding to Rprd1b. (**A**) In the Human Atlas database, the subcellular localization of SLC6A6 differs between A549 and U2OS cells: it is present in both the cell membrane and cytoplasm in one cell line, while showing slight expression in the nucleus in the other) (scale bar = 10 μm) (**B**) Immunohistochemical staining of PyMT tumor cells using two commercial Slc6a6 antibodies; the upper panel shows staining with the Abclonal antibody, and the lower panel with the HUABIO antibody (scale bar = 10 μm). (**C**) TNF-α treatment may promote the nuclear translocation of Slc6a6. The expression of the Slc6a6-GFP fusion protein is primarily localized to the membrane, with minor expression observed in the cytoplasm and cell nucleus (scale bar = 10 μm). (**D**) The protein interaction prediction of Slc6a6 and other proteins from GeneMANIA. (https://genemania.org/search/mus-musculus/Slc6a6) (Accessed on 2 September 2025). (**E**) The schematic of the tandem mass tag (TMT) 6plex-based quantitative proteomics (CO-IP/MS/MS) of SFB (Control cell) and Slc6a6-OE cells. (**F**,**G**) The intersection in the Venn diagram shows the potential proteins specifically interacting with Slc6a6. (**H**) The cell lysates were incubated with S beads. Five percent lysate was used as the input control. Blots probed with antibodies against the Flag and Myc epitope tags are shown, Flag refer Slc6a6 and Myc denote Rprd1b. (**I**,**J**) Optimal molecular docking model of Slc6a6 and Rprd1b predicted by the HDOCK server (UniProt ID: O35316 for Slc6a6 and UniProt ID: Q9CSU0 for Rprd1b). The pink structure represents Slc6a6, while the green structure corresponds to Rprd1b. (**K**) Pearson’s correlation found a positive correlation between SLC6A6 expression and RPRD1B by GEPIA (Gene Expression Profiling Interactive Analysis (http://gepia.cancer-pku.cn) (Accessed on 2 September 2025). (**L**,**M**) Co-transfection of SFB-Slc6a6 and Rprd1b-myc was performed in 293 T cells, with Myc serving as a marker for Rprd1b expression and flag for Slc6a6 expression by immunofluorescence and WB test. (**N**) Transient transfection of Si-RNA targeting Rprd1b into Slc6a6-OE cells followed by qPCR analysis after 24 h revealed that knockdown of Rprd1b reduced the expression levels of Slc6a6 and Ki67. Data are presented as mean ± SD, * *p* < 0.05, ** *p* < 0.01, *** *p* < 0.001.

Taurine is not incorporated into proteins because it contains a sulfonic acid group rather than a carboxylic acid group [62]. Whether an alternative mechanism exists—such as Slc6a6 interacting with intracellular proteins to promote tumor cell proliferation—rather than the effects being solely attributable to Taurine’s antioxidant activity. To address these questions, we first sought to determine the subcellular localization of Slc6a6. According to existing database reports, SLC6A6 is primarily localized on the cell membrane (Supporting Figure S3A). The Human Protein Atlas indicates that SLC6A6 is primarily localized to the cell membrane. Immunofluorescence staining images show that in the A549 lung cancer cell line, SLC6A6 is mainly distributed in the cell membrane, with expression also observed in the cytoplasm. In contrast, in the U2OS osteosarcoma cell line, SLC6A6 is localized within the nucleus (Figure 4A). However, reports on the nuclear expression of SLC6A6 are extremely limited [63]. We first examined the expression of Slc6a6 in PyMT tumors using antibodies; however, inconsistent results were obtained with different antibodies—some showed cytoplasmic expression, while others showed expression in both the cytoplasm and cell membrane, predominantly in the membrane (Figure 4B). To exclude potential antibody-related artifacts, we constructed a stable cell line expressing Slc6a6 fused with GFP (Slc6a6-GFP) to determine its subcellular localization. Our confocal fluorescence results showed that Slc6a6 is predominantly localized on the cell membrane, with a minor fraction present in the cytoplasm. Confocal fluorescence microscopy visualized the Slc6a6 in both cytoplasm and nucleus (Figure 4C). A study has reported that following TNF-α treatment, Slc6a6 immunolocalization is augmented, particularly in the nucleus and the perinuclear area [63]. Therefore, we investigated whether Slc6a6 could also be expressed in the nucleus of PyMT tumor cells under TNF-α stimulated conditions. Upon stimulation of Slc6a6-GFP cells with TNF-α, we observed a slightly stronger nuclear signal (Figure 4C). Given that protein function is largely determined by its subcellular localization, the nuclear presence of Slc6a6 suggests a potential functional role within the nucleus. To explore this further, firstly, we utilized bioinformatics databases to identify potential interacting partners of SLC6A6, but did not identify any proteins with known or potential associations with tumorigenesis or tumor progression (Figure 4D and Supporting Figure S3B–D). Then, we performed co-immunoprecipitation Mass Spectrometry (CO-IP/MS) experiments to identify potential interacting proteins with Slc6a6. By constructing SFB empty vector-transfected cells as a control, and comparing them with Slc6a6-SFB (Slc6a6 tagged with SFB), proteins were enriched and purified using S beads, digested, and subsequently analyzed by mass spectrometry (MS), after analysis and filtered, leading to the identification of 46 potential interacting proteins (Figure 4E–G). We systematically investigated the functions of these proteins through database annotation and literature review to narrow down the candidate list. Based on literature evidence, we selected eight proteins potentially associated with tumorigenesis (Ascl5, Ascl4, Itgb2, Pck2, Idh3g, Rab-8b, Txn2, Rprd1b) for further study. We individually cloned these eight candidate proteins and co-expressed them with Slc6a6 and a Myc-tagged with each candidate protein in 293T cells. Subsequent co-immunoprecipitation (Co-IP) experiments confirmed that Rprd1b as a binding partner (Figure 4H and Supporting Figure S3E). Previous studies have suggested that Rprd1b plays a role in cell cycle regulation [64,65]. Rprd1b, the regulation of nuclear pre-mRNA domain containing 1B gene, is a human homolog of the Rtt103 gene; it can regulate the binding of RNA polymerase II to the CCND1 gene (cyclin D1) and prevent degradation of the CCND1 mRNA [66,67]. Similarly, Rprd1b also enhances the transcription of many other cell cycle-related factors (such as CDK2, CDK4, CDK6 and cyclin E) [68,69,70]. Therefore, we hypothesized that the interaction between Slc6a6 and Rprd1b might promote cell cycle progression, thereby enhancing cell proliferation and tumor growth. The interaction between Slc6a6 and Rprd1b could represent a novel regulatory axis influencing cell cycle or transcriptional programs, independent of redox modulation. Furthermore, to test our hypothesis, we first employed protein docking to assess the potential interaction between the two proteins, followed by experimental validation. We downloaded the protein structures of Alfold from the UniProt database (Slc6a6-O35316 and Rprd1b-Q9CSU0) and performed molecular docking using HDOCK. Subsequently, we conducted visualization and graphical analysis of the three-dimensional complex model, while employing LigPlot+ to identify and present the two-dimensional interaction profiles. The top docking conformation with the highest interaction force scores were selected for display (Figure 4I,J). The docking results indicate that the two proteins exhibit high spatial complementarity within the binding pocket, enabling the formation of a stable complex under physiological conditions with significant binding affinity. The docking score of this interaction is −378.8, where a lower value indicates stronger binding, suggesting substantial thermodynamic stability of the complex (Supporting Figure S4A). In the protein–protein interaction, hydrogen bonds serve as the primary force maintaining complex stability. Multiple critical amino acid residues form hydrogen bonds, significantly enhancing the binding affinity between the proteins. Specifically, residues such as LYS-272 and LYS-244 of Rprd1b interact with Slc6a6 through hydrogen bonding (Figure 4J). These hydrogen-bonding interactions play a crucial role in stabilizing the tight association between Rprd1b and Slc6a6, not only consolidating the overall conformation of the complex but also potentially contributing to key regulatory functions during protein activity. In addition, non-covalent interactions, including electrostatic potential and hydrophobic interactions, are present within the complex, further reinforcing the compactness and binding strength at the interface (Figure 4I,J and Supporting Figure S4B–D). In summary, these docking results suggest that the protein–protein interaction has the potential to form a more intricate interaction network, providing structural evidence for its biological significance. Additionally, we observed a significant positive correlation between SLC6A6 and RPRD1B mRNA levels in breast tumors (R = 0.22, *p* = 5.7 × 10^−13^) (Figure 4K). Furthermore, Rprd1b and Slc6a6 through co-transfection and then subcellular fractionation experiments revealed the presence of both Rprd1b and Slc6a6 in the nuclear fraction, suggesting a potential interaction between the two proteins (Figure 4L,M). Given that previous studies have reported RPRD1B’s involvement in cell cycle regulation, we performed rprd1b knockdown and subsequently detected a decrease in the expression of the cell cycle marker like Ki67, CDH2 and CCNE1 by qPCR (Figure 4N and Supporting Figure S4E,F). Taken together, these findings suggest that regulation of Slc6a6 is associated with Rprd1b level in breast cancer. Importantly, our findings revealed that Slc6a6 may interaction with Rprd1b to exert its role in promoting cell cycle progression and proliferation.

### 3.5. The Expression Levels of Slc6a6 and Rprd1b May Serve as Indicators of Tumor Prognosis in Human Breast Cancer Cohort Analyses

**Figure 5:**
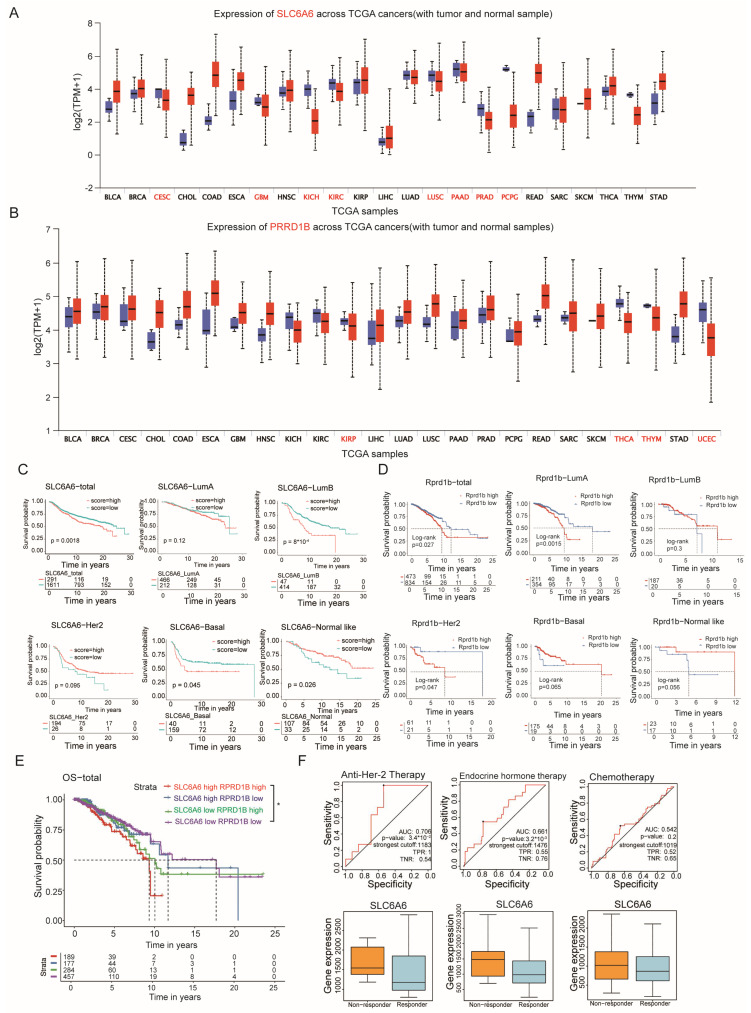
The expression levels of SLC6A6 and RPRD1B may serve as prognostic indicators for tumors in certain breast cancer subtypes, as suggested by human cohort analyses. (**A**) SLC6A6 expression levels were higher in most tumor tissues compared to normal tissues in the TCGA database (Black indicates high expression in tumors compared to normal tissues, while red indicates low expression in tumors compared to normal tissues). (**B**) RPRD1B expression levels were higher in most tumor tissues compared to normal tissues in the TCGA database (Black indicates high expression in tumors compared to normal tissues, while red indicates low expression in tumors compared to normal tissues.). (**C**) Prognostic analysis of SLC6A6 in different types of breast cancer based on Metabirc database. (**D**) Prognostic analysis of SLC6A6 in different types of breast cancer based on TCGA database. (**E**) Survival probability analyses in patients of survival probability in patients stratified according to the combination of SLC6A6 and RPRD1B based on TCGA database. (**F**) The ROC curve illustrating the validation of the diagnostic value of various therapies for breast cancer based on SLC6A6 expression, generated using the ROC-plotter tool within the Kaplan–Meier Plotter database and patient-derived data [71]. (Receiver operating characteristic (ROC) curves for SLC6A6. Values under the ROC curve ranges from 0.5 to 1. The closer the area under the curve (AUC) is to 1, the better the diagnosis. The horizontal coordinate is the False Positive Rate (FPR), and the vertical coordinate is the True Positive Rate (TPR).

To evaluate the translational relevance of our mouse model findings to human breast cancer, we analyzed bulk RNA sequencing data from breast cancer patients in the TCGA database. Consistently, SLC6A6 or RPRD1B is predominantly overexpressed in tumor tissues compared to normal tissues (Figure 5A,B), suggesting its oncogene potential in most cancers. We further examined the association between SLC6A6, RPRD1B, and clinical outcomes across molecular subtypes defined by PAM50 (Prediction Analysis of Microarray for 50 genes) classification. We first conducted separate analyses of SLC6A6 and RPRD1B with respect to prognosis. Our prognostic analysis revealed that for SLC6A6, high expression is associated with poor prognosis in most breast cancer subtypes like total, luminal B subtype and basal subtype. In contrast, for RPRD1B, high expression indicates poor prognosis only in total, luminal A, and HER-2 subtypes, whereas in other subtypes, the association is reversed (Figure 5C,D). When a dual-gene combined analysis of RPRD1B and SLC6A6 was performed on patients, it was found that patients with high expression of both SLC6A6 and RPRD1B exhibited an even worse prognosis (Figure 5E). Furthermore, we analyzed SLC6A6 in patients responding to different treatments for Relapse-free survival at 5 years and discovered that patients with higher SLC6A6 expression showed worse responses to anti-HER2 therapy (*n* = 50), endocrine hormone therapy (*n* = 907) and chemotherapy (*n* = 476) (Figure 5F). However, these results require further validation with larger patient samples, though they already provide us with some indications. These clinical data solidify SLC6A6-mediated Taurine transport as a driver of breast cancer malignancy and highlight its superior prognostic utility. Together, these data reveal the clinical involvement of SLC6A6 in breast cancer.

## 4. Discussion

Our data establish Slc6a6 as a significant prognostic biomarker and a potential therapeutic target. Clinical analyses demonstrate that high Slc6a6 expression is strongly associated with poor prognosis in breast cancer patients, underscoring its relevance in human cancer progression beyond preclinical models. Functional studies further validate its oncogenic role: Slc6a6 overexpression enhances Taurine uptake, conferring a proliferative advantage and reducing apoptosis in cancer cells. Additionally, Slc6a6 interacts with Rprd1b to promote cell cycle progression. Chronic stress induces systemic elevation of host Taurine levels, creating a Taurine-rich metabolic environment that tumors actively exploit through upregulation of Slc6a6. Intracellular Taurine functions not merely as a nutrient but as a potent signaling molecule that promotes cancer cell survival and proliferation. This mechanism may partially explain the poorer cancer outcomes observed in individuals experiencing chronic stress-tumors gain access to enhanced metabolic resources that support aggressive growth.

Previous studies have also observed increased Taurine levels in tumor-bearing mice, but these were merely descriptive without further functional exploration [72,73]. Our study provides more comprehensive insights. Although numerous studies have investigated Taurine, its role in tumorigenesis remains controversial [13,14,15,16,17,18]. One critical factor contributing to these inconsistent findings is the variation in Taurine concentrations used across studies. Here, we determined the physiological concentration of Taurine and conducted functional validation under these physiological conditions, thereby obtaining more accurate results. Furthermore, we discovered that Slc6a6 interacts with Rprd1b, a protein known to promote tumor progression, thus linking two proteins that were previously considered unrelated. Interestingly, SLC transporters are generally regarded as membrane proteins, yet we observed that Slc6a6 is expressed in the nucleus, suggesting that Slc6a6 may possess functions beyond substrate transport, potentially involving the regulation of gene expression. This observation is consistent with previous reports indicating nuclear localization of SLC6A6 [63]; however, no further studies have explored its nuclear expression or functions. This highlights the need for deeper investigation into the functional diversity of the SLC family of proteins. Notably, how can the increased expression of the Taurine transporter in tumor tissues be related to increased Taurine levels in blood? The relationship between transporter abundance in tumor cells and in the blood is influenced by numerous factors. In our study, we consider the following points: (1) The systemic metabolite levels in cancer are not solely determined by tumor consumption, but also by complex host-tumor interactions involving metabolic reprogramming, altered organ function (e.g., liver and kidney), and systemic stress responses [74]. In vivo, the systemic Taurine level appears to be influenced by multiple factors beyond tumor consumption alone, for example, increased whole-body Taurine production or release. It is possible that systemic upregulation of Taurine biosynthesis or tissue efflux outweighs tumor uptake, leading to net elevation in circulating Taurine. Secondly, compensatory systemic response has a role; the elevated plasma Taurine may represent a host compensatory mechanism to meet the increased demand from tumors and immune cells. Thirdly, regarding compensatory systemic response, the elevated plasma Taurine may represent a host compensatory mechanism to meet the increased demand from tumors and immune cells. Similar paradoxical increases in precursor metabolites have been reported in other contexts (e.g., elevated serum glutamine in some early-stage tumors despite high tumor consumption which is dependent on the tumor stage [75]). (2) The relationship between Slc6a6 and Taurine is not merely limited to uptake. While Taurine can be taken up by Slc6a6, Slc6a6 also possesses additional functions, such as its interaction with Rprd1b to promote proliferation. (3) Slc6a6 is expressed specifically within the stem cell population in tumors (we used the Cd200 marker to enrich for slc6a6-positive cells, since other studies have reported Cd200 as a marker for cancer stem cell [55,76]), which constitutes only a small proportion of the total tumor mass. As the tumor progresses, this proportion remains relatively stable; therefore, the levels of Taurine in the blood are unlikely to exhibit significant changes.

Our findings are consistent with prior studies underscoring the antioxidant properties of Taurine [77,78]. In this study, we demonstrate that Slc6a6 attenuates H_2_O_2_-induced oxidative damage by enhancing Taurine uptake, as evidenced by reduced reactive oxygen species (ROS) levels and increased glutathione (GSH) content in Slc6a6-overexpressing (Slc6a6-OE) cells. Notably, under basal conditions, Slc6a6-OE cells exhibited lower ROS levels and higher GSH concentrations, indicating that Slc6a6 overexpression improves basal redox homeostasis. This enhanced redox state likely contributes to the observed increase in basal cell viability, as cellular redox homeostasis-determined by a balance of redox processes and the activity of regulatory systems-is a critical factor in maintaining cell survival [79,80,81]. These broader nutritive and signaling roles of Taurine align with our subsequent findings on Rprd1b and cell proliferation, which likely represent a specific molecular pathway through which elevated intracellular Taurine-mediated by Slc6a6 overexpression-transmits pro-growth signals.

Nonetheless, this study has several limitations. The specific downstream signaling pathways activated by Taurine and their molecular interactions require further characterization. Moreover, the precise mechanisms by which stress hormones-such as cortisol and norepinephrine-regulate Slc6a6 expression, whether through direct transcriptional control or indirect signaling cascades, remain to be elucidated. Large-scale clinical validation is essential to confirm the robustness of our findings. Future studies should prioritize broader validation to reinforce and extend these results. assessing the clinical utility of serum Taurine levels and tumor Slc6a6 expression as biomarkers for identifying high-risk patients, predicting chemoresistance, or stratifying prognosis could yield significant translational impact.

In summary, we have identified that Slc6a6-driven Taurine uptake enhances cell fitness through a combination of improving cellular homeostasis (redox/nutrition) and activating a specific proliferative program via Rprd1b.

## Data Availability

This paper does not present original code or sequencing data. Raw mass spectrometry data are included in the Supplementary Materials. Any further information necessary to reanalyze the data reported in this study is available from the lead contact upon request.

## Supplementary Materials

The following supporting information can be downloaded at: https://www.mdpi.com/article/10.3390/cells15020207/s1, Figure S1: Quantitative results of Taurine in tumor-bearing and non tumor-bearing mice represented by mass spectrometry profiles. Figure S2: Slc6a6 may play a role in cancer, as indicated by data from our laboratory. (A) and (B) Slc6a6 expression levels and cell cycle activity in wild-type (WT) and K14-Cre Bcl11bfl/fl mice at 4 months of age (n = 9 per group). Data were derived from previously published results by Bai, H., Liu, X., Lin, M. et al. (2024) [37], which demonstrated that progressive senescence programs confer intrinsic susceptibility to aging-related female breast cancer (Nature Communications, 15, 5154). Figure S3: Based on database research results on SLC6A6-interacting proteins and localization (A) Prediction of subcellular localization of Slc6a6 the website Euk-mPloc 2.0 computation (accessed on 9 August 2024). (B) and (C) STRING protein–protein interaction (PPI) analyses for Slc6a6. Nodes represent the proteins required for interaction. Edges represent the associations between the proteins. The STRING web resource was used in the prediction of the PPI (Protein–Protein Interaction) network whereby an interaction score of >0.800 denoted a significant interactive relationship (accessed on 15 March 2024). (D) Protein interaction predictions for Slc6a6 and associated proteins, generated using GeneMANIA (https://genemania.org/search/mus-musculus/slc6a6), along with the detailed pathway representation (accessed on 15 March 2024). (E) The CO-IP data showed eight proteins potentially associated with tumorigenesis (Ascl5, Ascl4, Itgb2, Pck2, Idh3g, Rab-8b, Txn2, Rprd1b) by individually cloned these eight candidate proteins and co-expressed them with Slc6a6 and a Myc-tagged with each candidate protein in 293T cells. Figure S4: Rprd1b maybe binding with Slc6a6 (A) Docking results of the top nine candidate protein–protein complex conformations. The docking score indicates binding stability, with lower (more negative) values suggesting a more stable interaction. The top-ranked conformation has the lowest score among all results, indicating optimal binding energy. The confidence score reflects the reliability of the prediction, with values close to 1 indicating high reliability. Ligand RMSD (Å) measures the deviation of the ligand conformation from the reference conformation, where smaller values are preferable. (B) Overall structure of the protein–protein docking complex by HDOCK server. The pink structure represents Slc6a6, while the green structure corresponds to Rprd1b. (C) Electrostatic surface potential at the interface of the protein–protein docking complex. Color represents charge distribution: red regions indicate negative charge (acidic residues, such as Glu, Asp); blue regions indicate positive charge (basic residues, such as Lys, Arg, His); white regions represent neutral or hydrophobic areas. (D) Two-dimensional interaction diagram of the protein–protein docking complex (LigPlot+ analysis). Intuitively illustrates hydrogen bonds (green dashed lines), hydrophobic interactions (red semicircular arcs), and adjacent interactions of certain polar residues. (E) and (F) Transient transfection of three siRNA candidates targeting Rprd1b into Slc6a6-OE cells was performed. Following confirmation of knockdown efficiency, siRNA-1 was selected for further analysis. Quantitative PCR conducted 24 h post-transfection revealed that Rprd1b knockdown significantly reduced the expression of cell cycle-related genes CCND1 and CCNE1.
