# Supplementary material for: The Taurine-Slc6a6 Axis Promotes Breast Cancer Progression by Alleviating Oxidative Stress and Accelerating Cell Cycle Progression
Source: Cells. 2026 Jan 22;15(2):207. doi: 10.3390/cells15020207 (PMC12840004; doi:10.3390/cells15020207)
Supplement: Supplementary file 1 [file cells-15-00207-s001.zip › Supplementary Files/Support figures.pdf]

Figure S1

Taurine determination

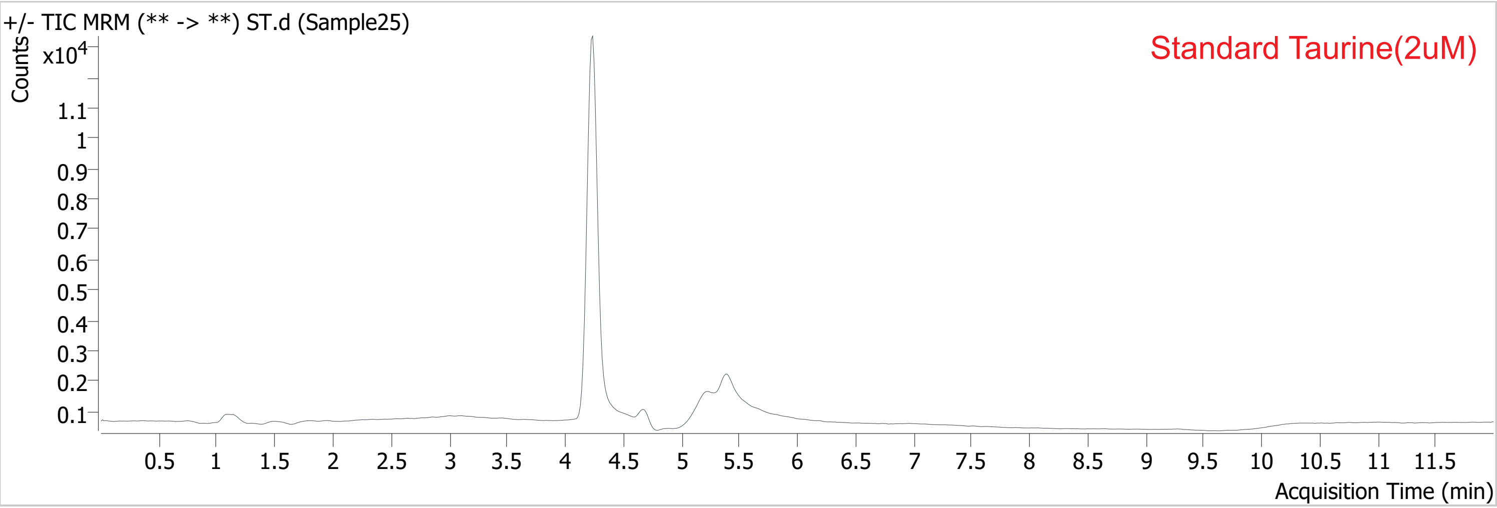

Compound	Transition	RT	Resp.	Final Conc	Units
Taruine	126.0 -> 44.1	4.235	66032	ND	ng/ml

Sample Chromatogram

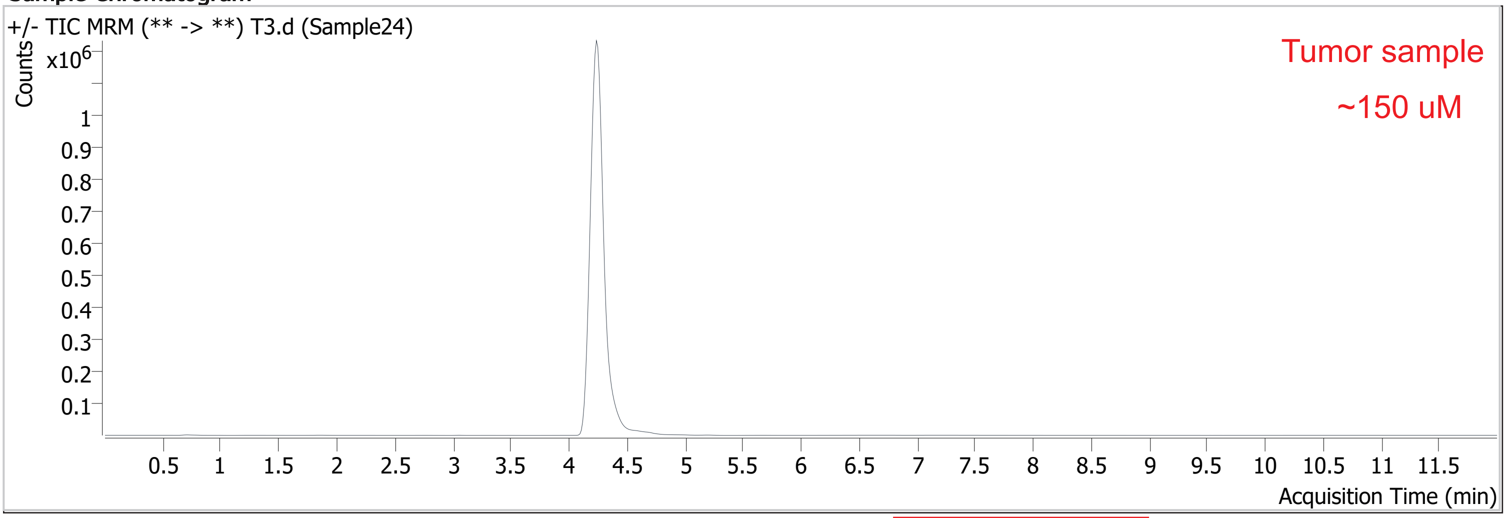

Compound	Transition	RT	Resp.	Final Conc	Units
Taruine	126.0 -> 44.1	4.235	4948030	ND	ng/ml

Taruine

Sample Chromatogram

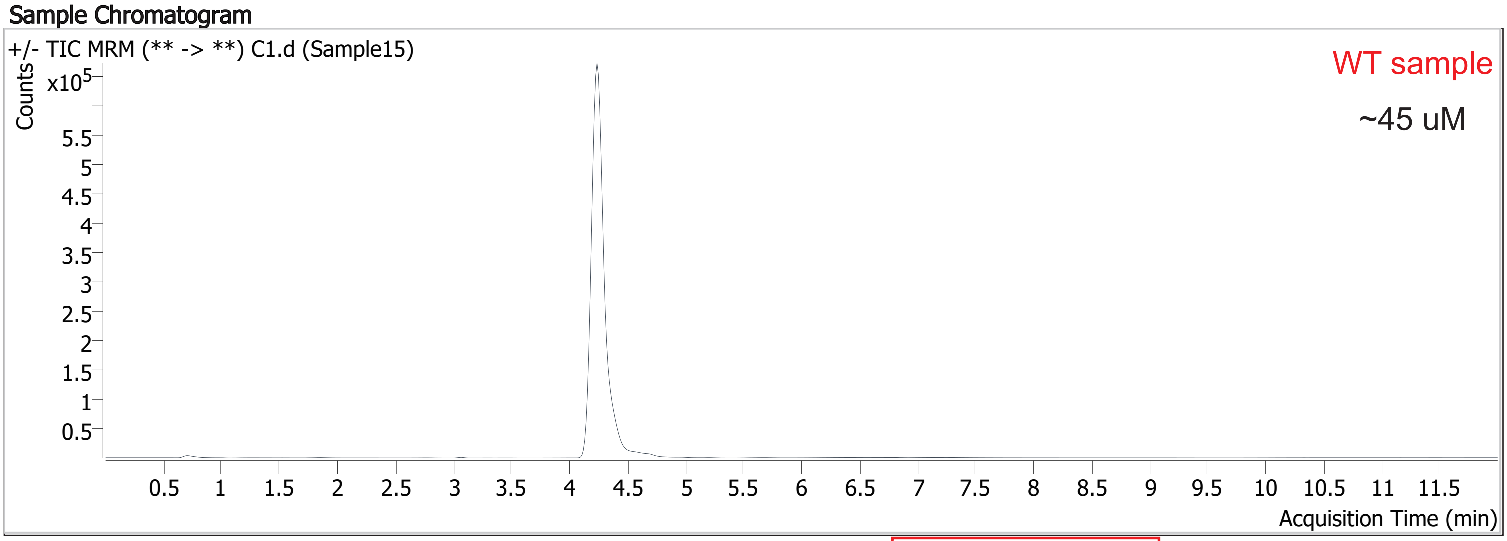

Compound	Transition	RT	Resp.	Final Conc	Units
Taruine	126.0 -> 44.1	4.235	2977014	ND	ng/ml

Figure S1 Related Figure 1

Quantitative results of Taurine in tumor-bearing and non-tumor-bearing mice represented by mass spectrometry profiles

Figure S2

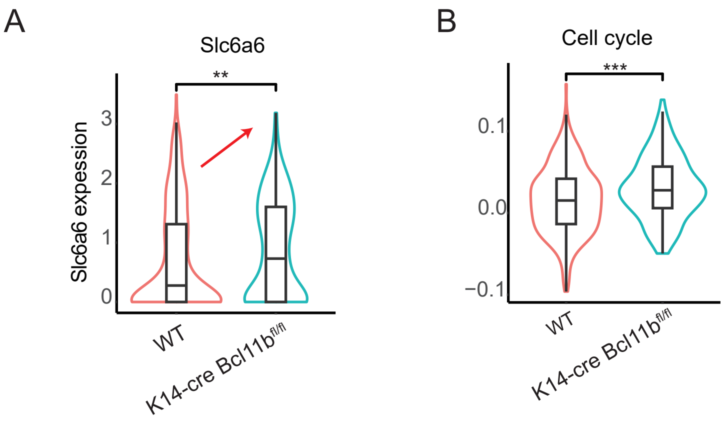
A
Slc6a6
**
Slc6a6 expession
3
2
1
0
WT
K14-cre Bcl11b$^{fl/fl}$
B
Cell cycle
***
0.1
0.0
−0.1
WT
K14-cre Bcl11b$^{fl/fl}$

Figure S2 Related Figure 2

(A) and (B) Slc6a6 expression levels and cell cycle activity in wild-type (WT) and K14-Cre Bcl11bfl/fl mice at 4 months of age (n = 9 per group). Data were derived from previously published results by Bai, H., Liu, X., Lin, M. et al. (2024), which demonstrated that progressive senescence programs confer intrinsic susceptibility to aging-related female breast cancer (Nature Communications, 15, 5154).

Figure S3

A

Euk-mPloc 2.0 Computation Result

Query Protein	Predicted location(s)
sp|O35316|SC6A6_MOUSE Sodium- and chloride-dependent taurine transporter	Cell membrane

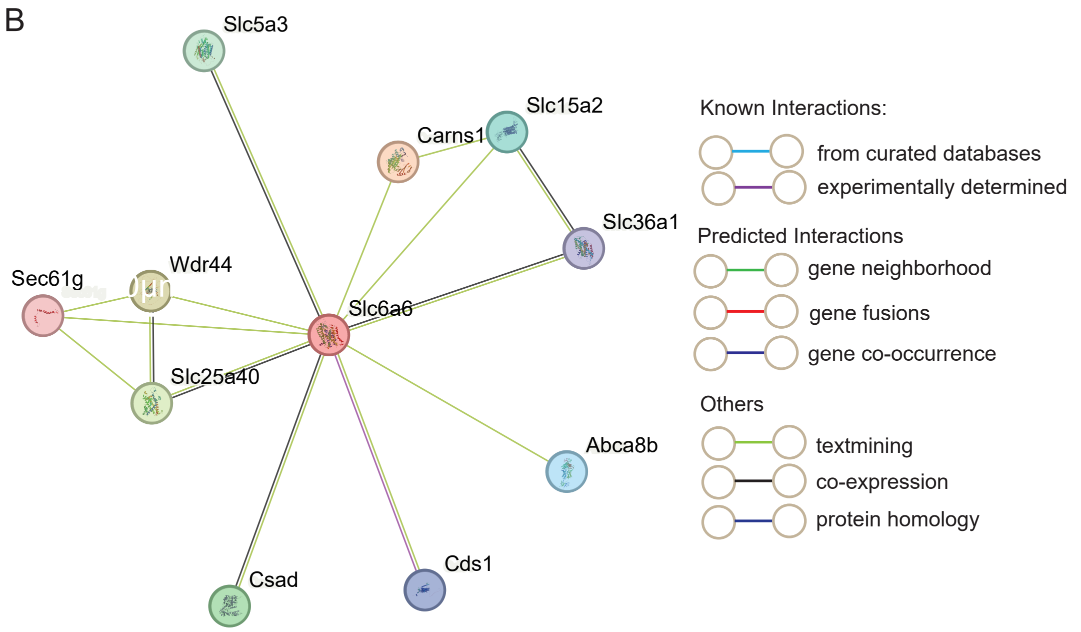

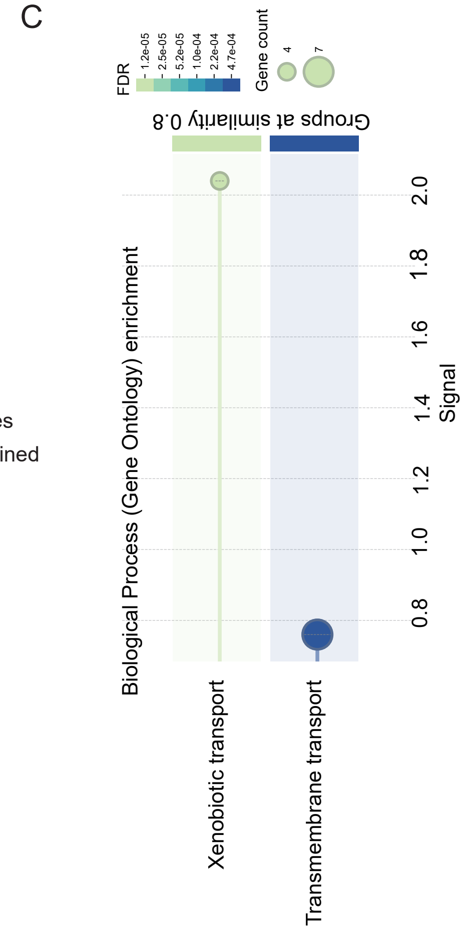

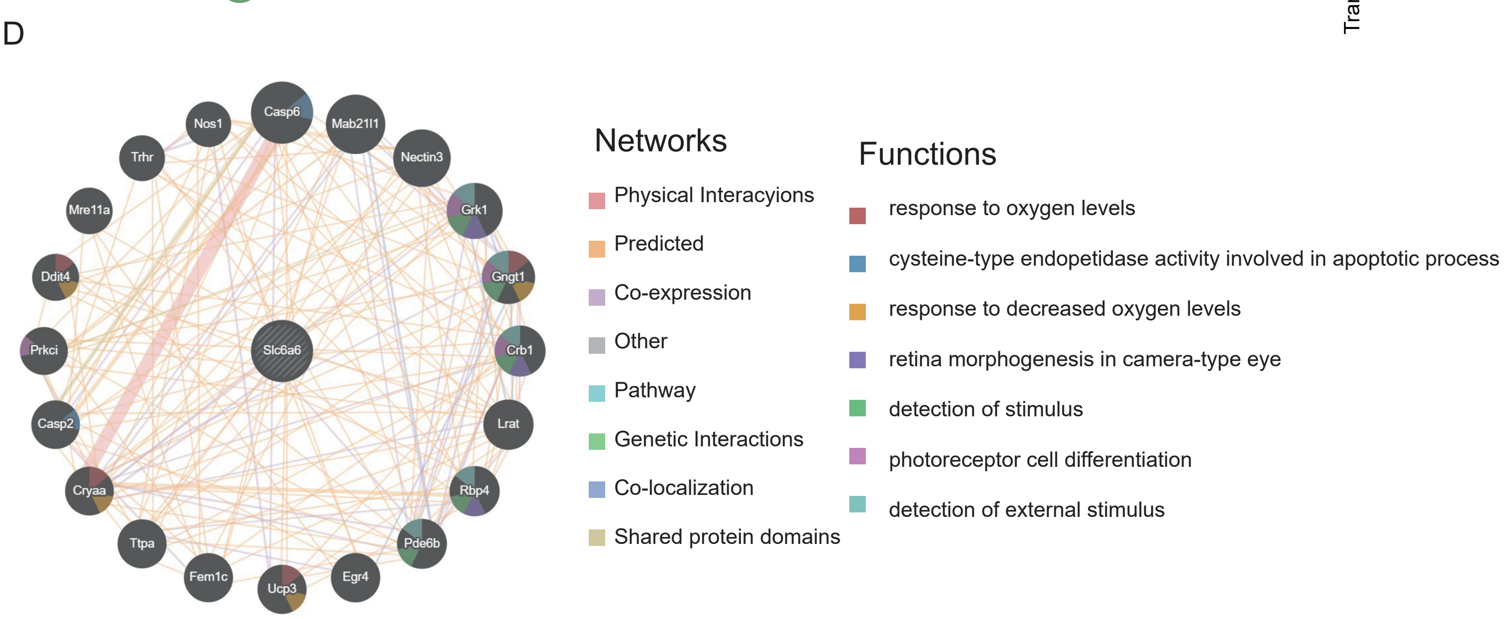

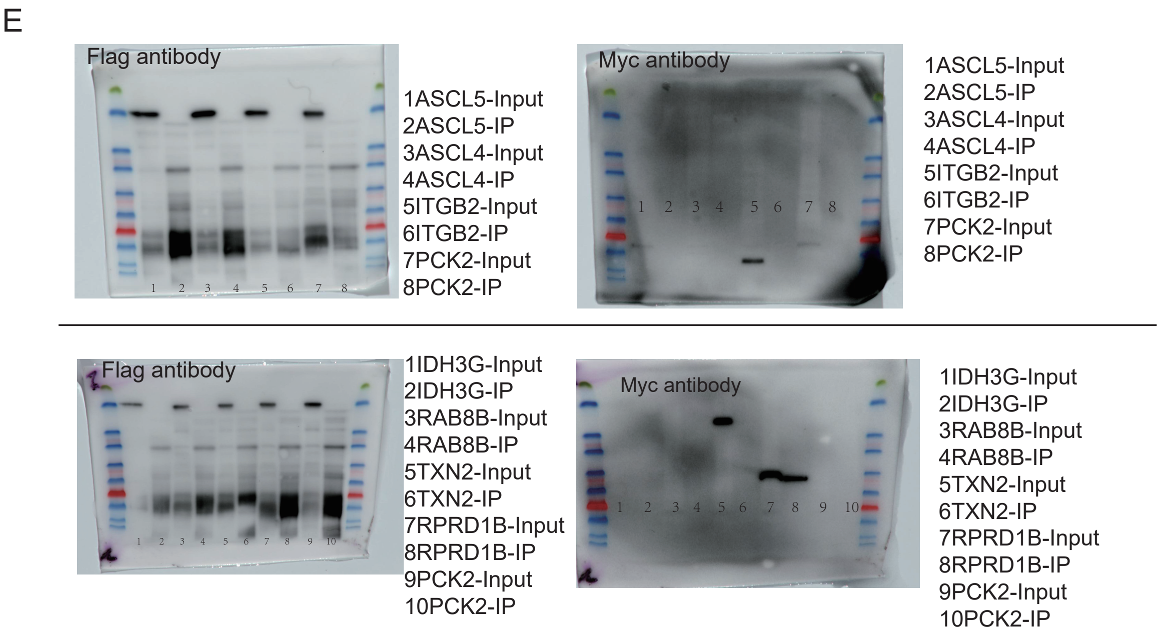

Figure S3 Related Figure 4

(A) Prediction of subcellular localization of Slc6a6 the website Euk-mPloc 2.0 computation.

(B) and (C) STRING protein–protein interaction (PPI) analyses for Slc6a6. Nodes represent the proteins required for interaction. Edges represent the associations between the proteins. The STRING web resource (http://www.stringdb.org) was used in the prediction of the PPI (Protein–Protein Interaction) network whereby an interaction score of >0.800 denoted a significant interactive relationship.

(D) Protein interaction predictions for Slc6a6 and associated proteins, generated using GeneMANIA (https://genemania.org/search/mus-musculus/slc6a6), along with the detailed pathway representation.

(E) The CO-IP data showed eight proteins potentially associated with tumorigenesis (Ascl5, Ascl4, Itgb2, Pck2, Idh3g, Rab-8b, Txn2, Rprd1b) by individually cloned these eight candidate proteins and co-expressed them with Slc6a6 and a Myc-tagged with each candidate protein in 293T cells.

Figure S4

A

Table. Docking results of the top nine candidate protein–protein complex conformations.

Rank	Docking SCore	Confidence Score	Ligand rmsd(Å)
1	-378.8	0.9898	61.92
2	-366.6	0.987	63.34
3	-319.6	0.9675	32.2
4	-310	0.9608	60.95
5	-307.2	0.9587	52.2
6	-301.4	0.9538	68.47
7	-300	0.9526	60.22
8	-299.5	0.9521	68.01
9	-297.7	0.9505	57.24

C

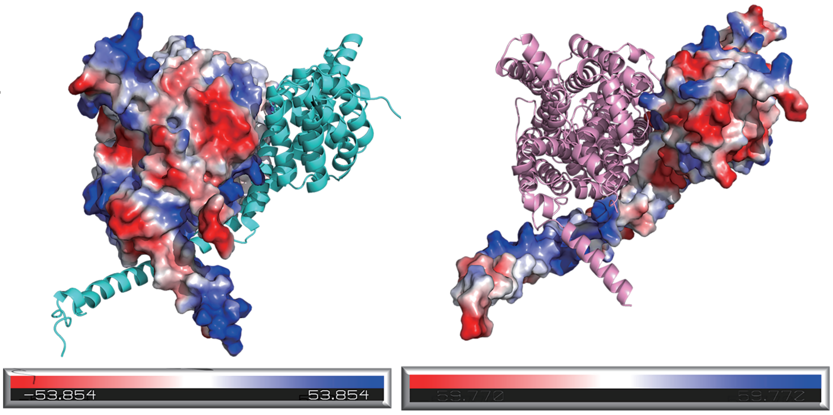

B

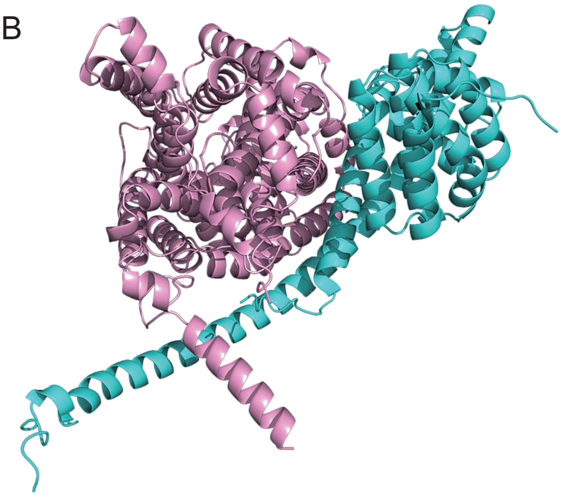

D

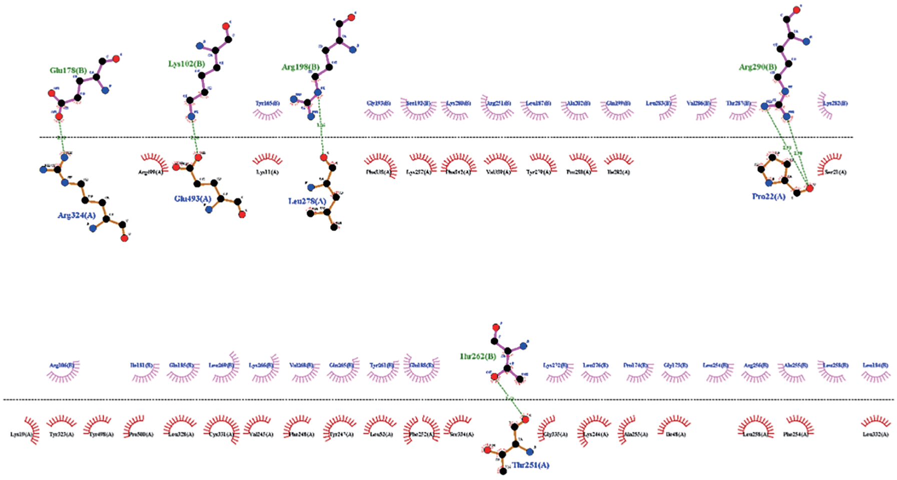

E

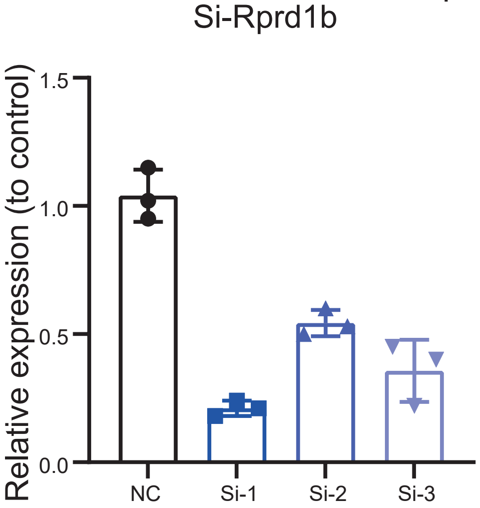

F

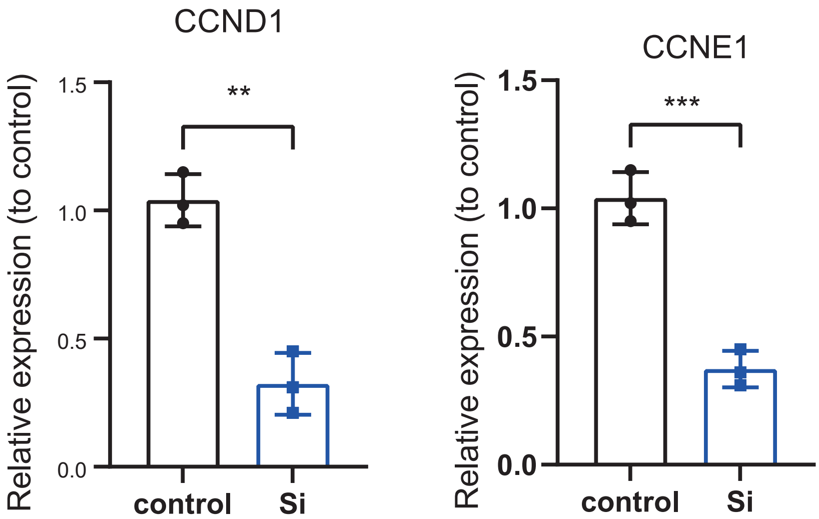

Figure S4 Related Figure 4.

(A) Docking results of the top nine candidate protein–protein complex conformations.The docking score indicates binding stability, with lower (more negative) values suggesting a more stable interaction. The top-ranked conformation has the lowest score among all results, indicating optimal binding energy. The confidence score reflects the reliability of the prediction, with values close to 1 indicating high reliability. Ligand RMSD (Å) measures the deviation of the ligand conformation from the reference conformation, where smaller values are preferable.

(B)Overall structure of the protein–protein docking complex by HDOCK server. The pink structure represents Slc6a6, while the green structure corresponds to Rprd1b.

(C)Electrostatic surface potential at the interface of the protein–protein docking complex. Color represents charge distribution: red regions indicate negative charge (acidic residues, such as Glu, Asp); blue regions indicate positive charge (basic residues, such as Lys, Arg, His); white regions represent neutral or hydrophobic areas.

(D)Two-dimensional interaction diagram of the protein–protein docking complex (LigPlot+ analysis). Intuitively illustrates hydrogen bonds (green dashed lines), hydrophobic interactions (red semicircular arcs), and adjacent interactions of certain polar residues.

(E) and (F)Transient transfection of three siRNA candidates targeting Rprd1b into Slc6a6-OE cells was performed. Following confirmation of knockdown efficiency, siRNA-1 was selected for further analysis. Quantitative PCR conducted 24 hours post-transfection revealed that Rprd1b knockdown significantly reduced the expression of cell cycle-related genes CCND1 and CCNE1.
