# Supplementary material for: The Taurine-Slc6a6 Axis Promotes Breast Cancer Progression by Alleviating Oxidative Stress and Accelerating Cell Cycle Progression
Source: Cells. 2026 Jan 22;15(2):207. doi: 10.3390/cells15020207 (PMC12840004; doi:10.3390/cells15020207)
Supplement: Supplementary file 1 [file cells-15-00207-s001.zip › Supplementary Files/original WB figures/original WB for Figure 4M.pdf]

1. The WB original, uncropped and unadjusted images for Figure 4M

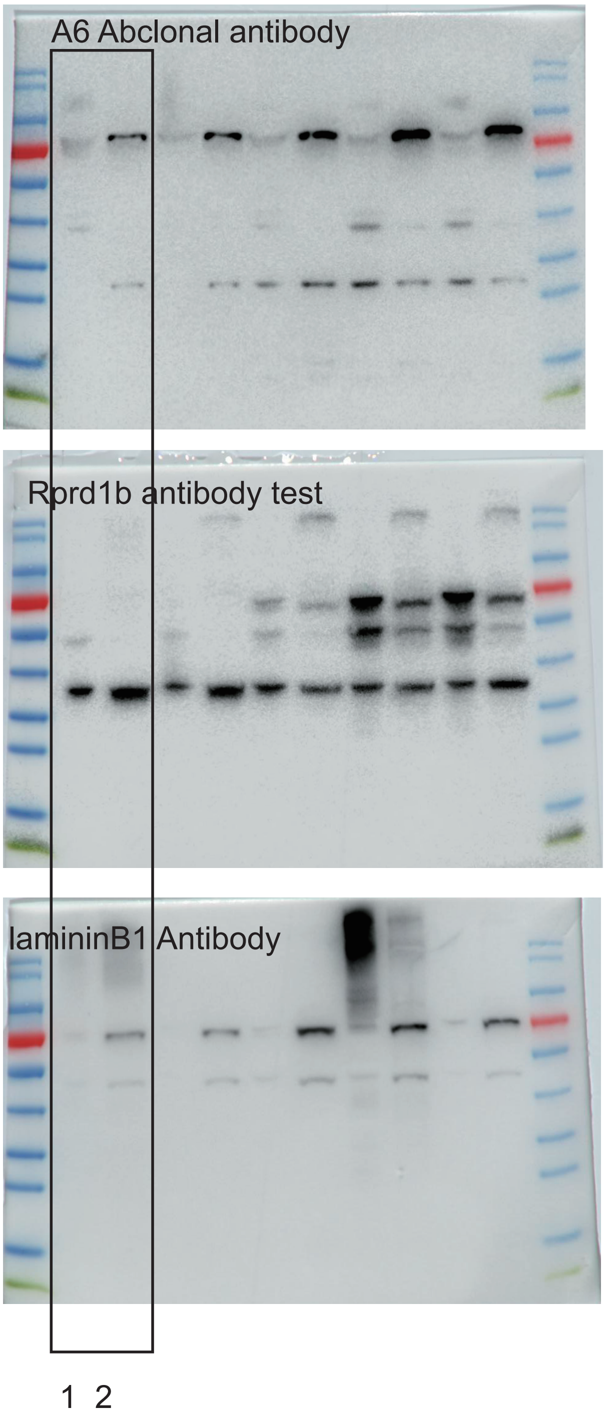

Lane 1 indicated cytoplasmic components
Lane 2 refered cellular nuclear components
