# Supplementary material for: The Taurine-Slc6a6 Axis Promotes Breast Cancer Progression by Alleviating Oxidative Stress and Accelerating Cell Cycle Progression
Source: Cells. 2026 Jan 22;15(2):207. doi: 10.3390/cells15020207 (PMC12840004; doi:10.3390/cells15020207)

1. The WB original, uncropped and unadjusted images for Figure 4H

Flag antibody(slc6a6)

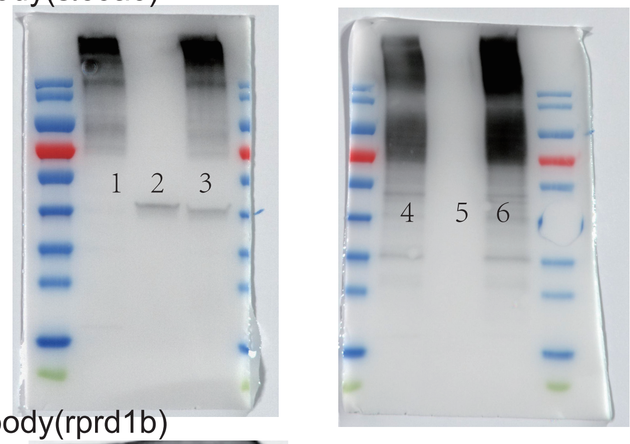

123 Input sample

1 slc6a6-flag-input
2 rprd1b-myc-input
3 slc6a6-flag+rprd1b-myc-input

456 IP sample

4 slc6a6-flag-ip
5 rprd1b-myc-ip
6 slc6a6-flag+rprd1b-myc-ip

MYC antibody(rprd1b)

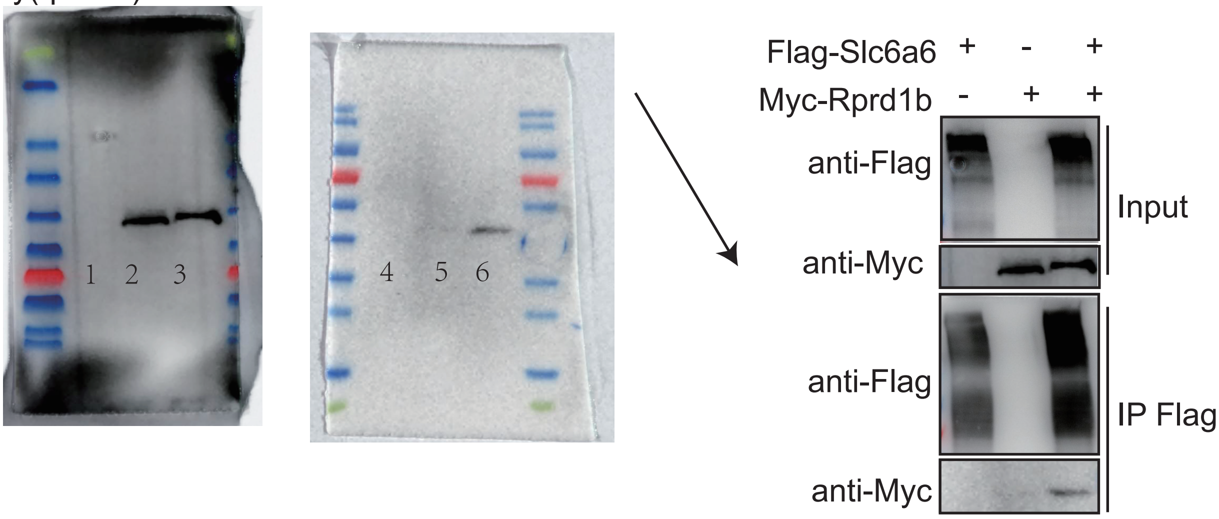

Supplement: Supplementary file 1 [file cells-15-00207-s001.zip › Supplementary Files/original WB figures/original WB for Figure 4H.pdf]
